# The Konark Temple’s Construction: A Critical Review of the Historical, Cultural, and Scientific Evidence

**DOI:** 10.12688/f1000research.157831.1

**Published:** 2024-12-20

**Authors:** Susanta Bahinipati, Dipti Ranjan Biswal, Damodar Suar

**Affiliations:** 1 School of Film and Media Sciences, Kalinga Institute of Industrial Technology, Bhubaneswar, Odisha, 751024, India; 2 School of Civil Engineering, Kalinga Institute of Industrial Technology, Bhubaneswar, Odisha, 751024, India; 3 School of Liberal Studies, Kalinga Institute of Industrial Technology, Bhubaneswar, Odisha, 751024, India

**Keywords:** Konark Temple, Construction Techniques, Historical Significance, Sundial, Astronomy, Indigenous Knowledge

## Abstract

Drawing from historical accounts, cultural practices, archaeological discoveries, and indigenous technical knowledge, the research presents a critical review of varied aspects of the construction of the Konark temple and presents future research directions. To fulfill the purpose, documents were sourced from Scopus, Google Scholar, and ancient texts and palm leaves. Findings suggest the legacy of Narasimhadeva I, the Hindu monarch who remained undefeated during his reign, safeguarded the kingdom from encroaching Muslim rulers. The narrative highlights the bravery of a faithful elephant that revived the king on the battlefield. The Konark temple was built with the dual purpose of showcasing the glory of his victory and devotion to the Sun God. The ancient artisans’ knowledge, skills, and commitment were crucial in erecting this tallest temple structure. The investigation further illuminates the use of sea routes for transporting monumental stones, the ingenuity in laying the temple’s foundation, the selection of high-grade stones, the monumental task of lifting colossal stones, like the world’s heaviest stone hoisted to a height of about two hundred feet, the use of rust-resistant iron, and the application of advanced astronomical knowledge by ancient artisans. The study provides insights into ancient engineering ingenuity, encouraging further exploration of the enduring legacy of the Konark temple’s construction.

## Introduction

The Sun Temple of Konark, also known as the Konark Temple (KT) in Odisha, stands out among the multitude of exquisitely crafted temples spread throughout India. It has fascinated countless generations with its remarkable magnificence, radiance, splendor, and enigma. The KT is included in the list of seven wonders of India and the list of World Heritage monuments, as designated by UNESCO in 1984 ( Dey, 2016; Donaldson, 2003; Jana *et al.*, 2016). The KT with 19.8921° N, 86.0912° E, is situated 64 kilometers in the southeast direction from Bhubaneswar, and 35 kilometers in the northeast direction from the city of Puri along the coastline of the Bay of Bengal. The temple’s construction was initiated by the king, Narasimhadeva I, known as Langula Narasimhadeva, during the Eastern Ganga dynasty ( Behera, 2005; Bussagli, 1971; Chakravarti, 1904; Chauley, 1997; Das, 2015; Dey, 2016; Donaldson, 1985; Edwards, 1969; Eschmann, *et al.*, 1978; Ganguly, 1912; Jana, *et al.*, 2021; Joshi & Srivastava, 2021; Mahapatra, 1989; Mohanty, 2001; Percy, 1959; Rowland, 1953; Swarup, 1910). The temple, dedicated to *Surya Deva* (Sun God), was constructed in the mid-thirteenth century. Despite significant deterioration, the remaining portions of the temple now exhibit the delicate craftsmanship of that historical period.

The construction of the temple began around the fifth *anka* (regnal year) of Narasimhadeva I’s rule, employing 1200 workers ( Behera, 2005; Boner *et al.*, 1972; Das, 2011; Dey, 2016; Donaldson, 1985; Mahapatra, 1989; Mishra, 1919; O’Malley, 1908). It took about 12 years, 10 months, and 14 days to finish, completing in the 18th *anka* of Narasimhadeva I’s reign ( Dey, 2016; Donaldson, 1985). The temple was sanctified on a Sunday, namely on the day of *Magha Saptami* (seventh day in a lunar fortnight of January or February month), which fell on the 27th of January in the year 1258 A.D. ( Dey, 2016; Donaldson, 1985). The occurrence of *Magha Saptami* on a Sunday is regarded as propitious and this event takes place just once in seven years which is also called *Vijaya saptami* (seventh day of triumph) ( Swarup, 1910). Narasimhadeva I desired to consecrate the temple on a highly favorable day, and hence he persistently urged the *Sutradhara* (master architect) to finish the work within the planned timeframe.

Studying the ancient temple of Konark is imperative for the techniques it embodies, which continue to influence modern architectural and engineering practices. Experts’ studies undertaken by the Council of Scientific and Industrial Research (CSIR), Central Building Research Institute (CBRI), and the Indian Institute of Astrophysics (IIA), demonstrate the sophistication and ingenuity of ancient craftsmanship. Analyzing the architectural feats of the KT, researchers gain insights to integrate ancient techniques with modern structures. It was evident in the Ram Temple in Ayodhya on 17 April 2024. The direct sunlight casting a ‘Surya tilak’ on Ram’s forehead facing east at specific times during *Ram Navami* (celebration of Lord Ram’s birth on ninth day of Chaitra: March or April) symbolizes the fusion of ancient KT’s technical knowledge with modern innovations. Furthermore, an exploration of the techniques used to transport and lift massive stone sculptures, along with the discovery of rust-resistant iron beams within the KT compound, showcases the engineering prowess of ancient times and emphasizes the importance of delving deeper into the construction methods and artisanal craftsmanship. The presence of colossal sculptures that have endured for generations also underscores the necessity of conducting comprehensive studies to uncover the intricacies involved in such aspects of KT.

Beneath the KT grandeur lies a tapestry woven with controversies, myths, and scholarly debates, each thread contributing to the intricate narrative surrounding its construction. We examine the temple’s construction purpose, the socio-economic conditions of the kingdom at the time, and the temple’s original dimensions obscured by its ruined state upon excavation in the early twentieth century. We further scrutinize the folk legends surrounding the temple and its completion, stone selection, transportation of large stones from distant quarries, and the indigenous technology used in constructing this colossal structure.

**Figure 1.**
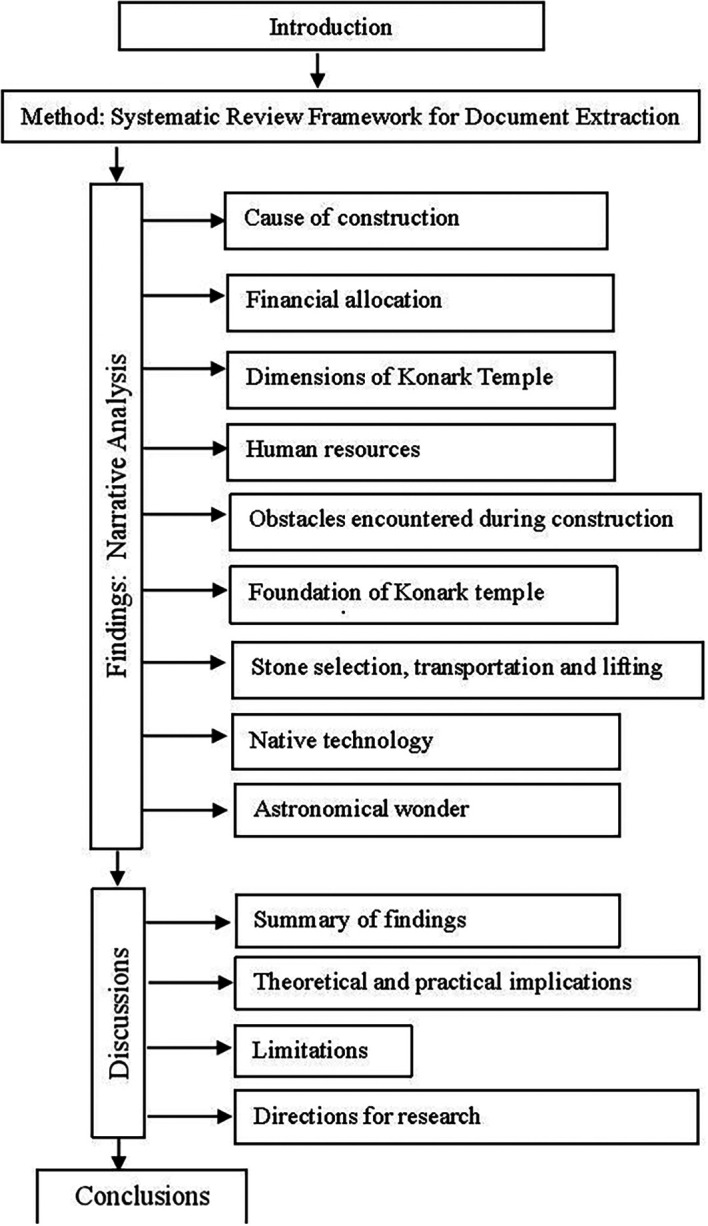
Structure of the paper (Source: Authors).

Against this backdrop, the study aims to present a critical review of the historical, cultural, and scientific evidence from past to present to provide a full account of the KT construction. These three aspects are not mutually inclusive. The structure of the paper is shown in Figure 1. Next, it mentions the sourcing of documents in the method section, followed by the reporting of the findings from the narrative inquiry on each of the nine issues. Then, the discussion section summarizes the findings, mentions theoretical and practical implications, limitations, provides directions for research, and concludes with a novel contribution to knowledge ( Figure 1).

## Method

**Figure 2.**
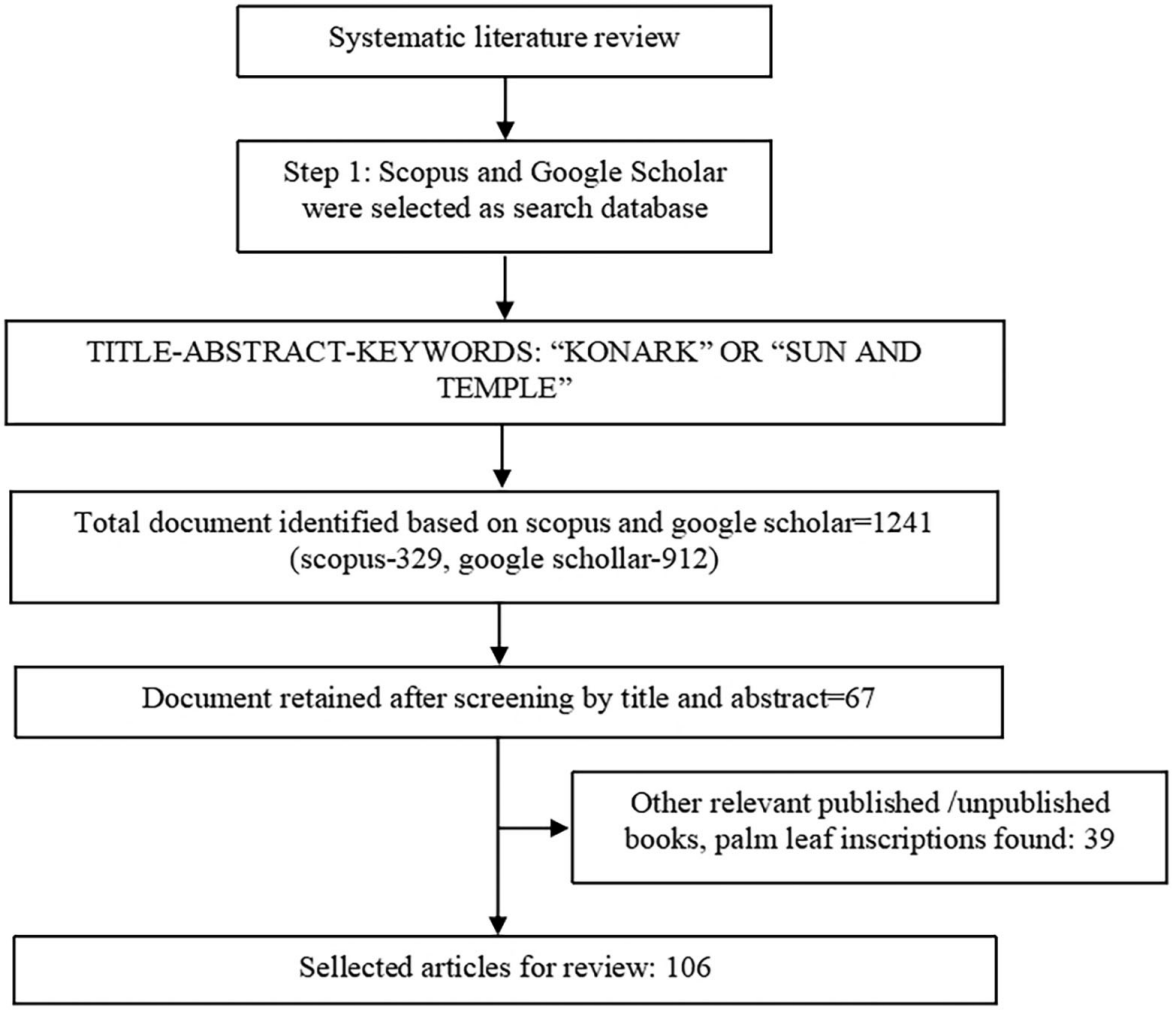
Systematic review framework for extraction of documents (Source: Authors).

A systematic review methodology was adopted for documents search and selection. Documents were extracted from Scopus, the largest scientific database of journals, and Google Scholar, the automated, inclusive database. Those yield maximum access to published documents ( Bar-Ilan, 2008). Documents were extracted using search strings “KONARK” OR “SUN AND TEMPLE” in title, abstract, and keywords. A total of 329 documents were extracted from Scopus and 912 were extracted from Google Scholar. Seventy documents relevant to the purpose of the study and related to architecture, construction, water bodies, and folklore connected to the KT were retained. As KT is an ancient structure, many documents appeared in books and ancient informal writings were considered relevant, 39 books and ancient writings were thoroughly read to satisfy evidence requirements. Figure 2 presents a flowchart outlining the process for selecting papers for a comprehensive review. The narrative analysis of these documents is reported in the next section.

## Findings

### Cause of construction

The KT was built with the dual purpose of expressing devotion and gaining fame. Narasimhadeva I is acknowledged in the field of art, thereby earning him the title of *‘Shilpajna’* (knowledgeable in sculpture), elaborated in Ekabali, written by Vidyadhara a Sanskrit poet of 13 ^th^ century ( Chakravarti, 1904; Donaldson, 2003; Mahapatra, 1959). The monument is believed to be a memorial dedicated to commemorating his military campaigns ( Behera, 2005; Boner *et al.*, 1972; Bussagli, 1971; Dey, 2016; Donaldson, 1985; Edwards, 1969; Ganguly, 1912; Jarrett, 1891; Joshi & Srivastava, 2021; Mahapatra, 1959; Mahapatra, 1989; Subbarao, 1939). The construction of the monument began shortly after his military triumph, and the campaigns persisted until the completion of the construction. The war concluded with his remarkable victory over the Muslim rulers ( Banerji, 1930; Boner *et al.*, 1972; Chinnappa, 1978; Donaldson, 2003; Panigrahi, 1986; Sarkar, 2003; Subbarao, 1939). This is manifested in several ornamental sculptures depicting war horses, diverse combat actions, and notably over 1,700 elephants ( Donaldson, 2003), thereby substantiating the title *Gajapati* (Lord of elephants) ( Eschmann *et al*., 1978; Fabri, 1974; Kulke, 1993: Kulke & Rothermund, 2016). As per the inscription of the Kapilash temple, Dhenkanal, Odisha, of 1246 A. D, he was the first king of Odisha to be bestowed the title of Gajapati, with a huge troop of elephants in his army. Few sculptures in the KT were created by soldiers who took part in the conflict. It depicts the firsthand experience of the victorious and joyful moment ( Boner *et al.*, 1972). In a separate palm leaf document, namely XLVII.20 ( Boner *et al.*, 1972), *Sutradhara* requested a skilled warrior as well as a sculptor to create a monument depicting the act of ‘Suasamala fighting’ while mounted on a horse. Observing the pair of life-size war horses at the south gate of the KT, an European art historian, E. B. Havell, expressed that the Indians showcased their pride in victorious warfare through the immense strength depicted in these sculptures, comparable to the fiery and passionate expressions found in the greatest European art ( Havell, 1908). Upon observing the deteriorated condition of the impressively grand statue, he expressed that if this exceptional work of art were identified as ‘Greek’ or ‘Roman’, it would have been exhibited in a renowned museum in America or Europe ( Behera, 2005; Dey, 2016; Ganguly, 1912; Havell, 1908). It is understood that Indians in ancient times possessed exceptional abilities to comprehend and effectively utilize their creative skills in harnessing the natural resources of their homeland.

In antonymous, a pair of galactic elephants standing at the north gate, there is a great memory of king Narasimhadeva I lying behind the elephant sculptures; which has been mentioned in the *Madalapanji* (a chronicle of Jagannath temple) ( Boner *et al.*, 1972; Dey, 2016). It was all about a day in the war field at Sasipur, when King Narasimhadeva I was stuck and got unconscious. Everyone on the battlefield searched for the dead body, suspecting the king had been killed. In the meantime, a small elephant of the king, named Sudehi, carried him up to the camp with its trunk and saved his life. From that day, Narasimhadeva I liked him as his son; in consequence, a special team had been allocated responsibility to make a sculpture of Sudehi with the instruction that he should look real; the intention of the king was to immortalize him for all times.

Mark Roland Shand, a travel writer and conservationist (BBC) visited the KT on the back of an elephant named Tara. An interesting incident happened in front of the pair of elephants at the north gate when Tara performed “her ceremonial ‘ *pranam*’ by lifting her trunk in salute” ( Shand, 1992). Appreciating the pair of elephant sculptures he said that the colossal war elephant would be mistaken by the visitors on a moonlit night as real. Not only war and struggle have been portrayed in the decorated sculpture: there are vast varieties of the endless richness of life, living, profession, approach, originality, culture, music, sensuous themes and nymphs. Every part of the temple exterior is ornamented with details, endless varieties of work carved with minute detail, it is said that “Indians plan like titans, finish like jewels”. Even, Ananda Coomaraswami states that “it would be hard to find anywhere in the world a more perfect example of the adaptation of sculpture to architecture” ( Coomaraswamy, 1913, p.75). The KT is an epic, where eternal truth had been encrypted in the form of art defying time through chiseling the stones.

### Financial allocation

Abu’l Fazl commented in Ain-i-Akbari, suggesting that the king allocated the revenue of twelve years of the kingdom for the completion of the construction of KT ( Jarrett, 1891). According to historical records, during his reign, Kalinga (ancient name of Odisha) experienced a period of great wealth. Contrarily, Abu’l Fazl acknowledges this fact by stating that it is a “mighty memorial to posterity” ( Donaldson, 2003; Jarrett, 1891). Before ascending to the throne, he led the war as a commander and achieved numerous victories, which enabled him to amass substantial wealth. Returning from war, his mother proposed to utilize his newfound riches to construct a Sun temple in Konark ( Dey, 2016; Donaldson, 1985) as a sacred place, like Biraja temple in Jajpur, Lingaraj temple in Bhubaneswar, and Jagannath temple in Puri, that was lagging with a supper structure. According to the *Madalapanji*, Anangabheemadeva III regularly approved annual budgets for the daily worship and upkeep of the temple, considering the offerings made during the puja ceremonies, the Seven *Dhupas*, as well as all the rituals, festivals, and special events. Anangabheemadeva III raised the sanction from 42,500 *Kahanas* (cowrie) to 52,000 *Kahanas* ( Dey, 2016; Donaldson, 2003; Swarup, 1910). Additionally, the kingdom’s currency was changed from *Kahana* to gold coin, with 15 *Kahanas* being equivalent to 1 gold coin. This change symbolizes the kingdom’s wealth and prosperity.

### Architectural design

The project commenced during the reign of Narasimhadeva I, when Sadasiva Samantaraya, the second minister of King Anangavimadeva III, was appointed as the chief architect for the construction ( Mishra, 1919; Mohanty, 2001). The preliminary tasks, consisting of drafting blueprints, extracting stones from quarries, and hiring labourers, lasted nearly six years ( Behera, 2005; Dey, 2016; Donaldson, 2003; Mahapatra, 1989; Swarup, 1910).

**Figure 3.**
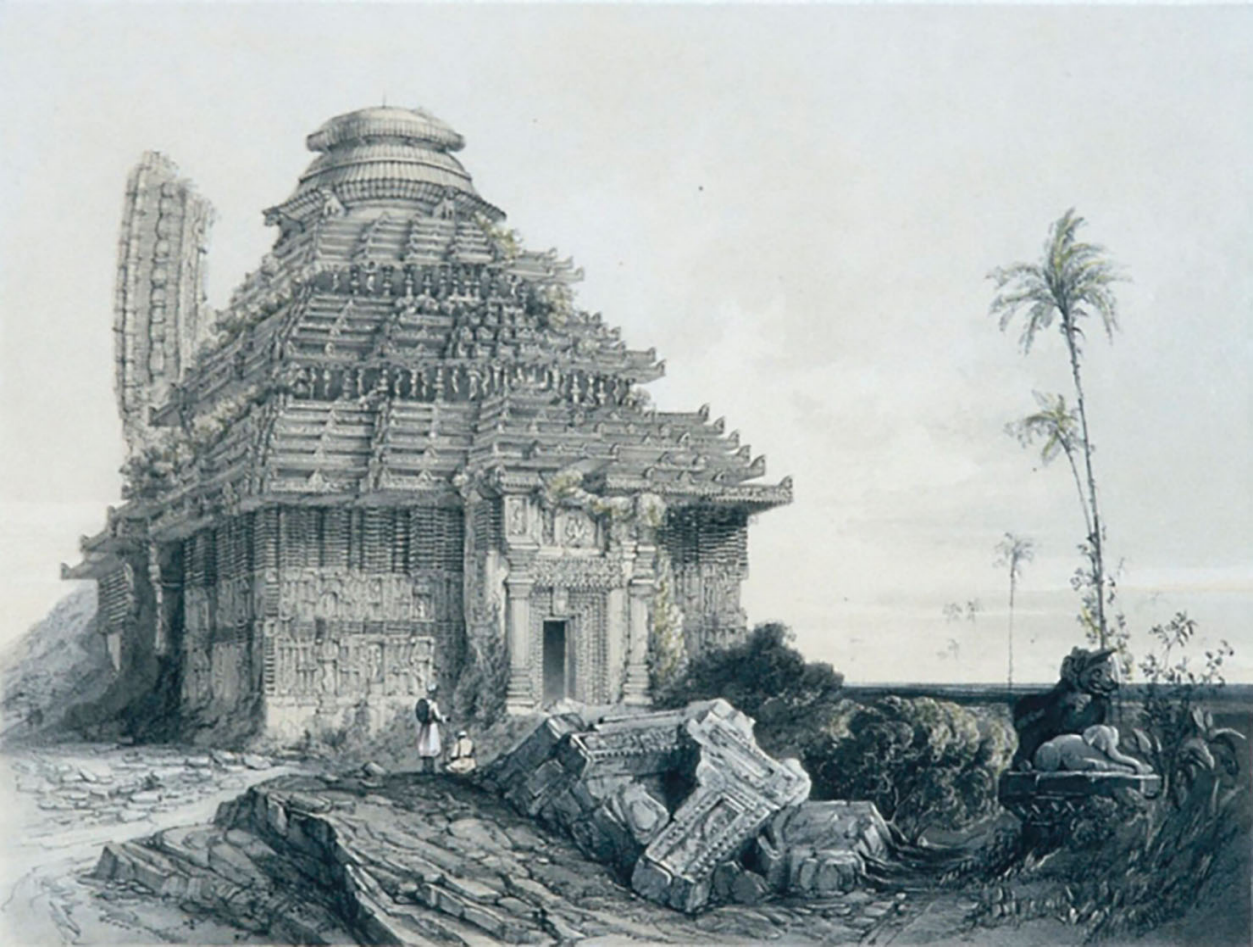
The 3D view of KT ( Fergusson, 1848).

In 1837, James Fergusson visited the temple and created a drawing of the KT ( Figure 3), which provided a clear depiction of the temple’s physical condition at that time. According to Fergusson (1848), the height of the sanctum in the painting is around 140 to 150 ft, which is taller than *Jagamohana* (porch). The KT is encompassed by boundary walls measuring 261.2m (857 ft) by 164.6m (540 ft) ( Donaldson, 2003; Edwards, 1969; Ganguly, 1912), with a height of 14 ft and thickness of 5 ft and 4 in ( Ganguly, 1912). The architectural style of the Orissan temples (Kalinga style of architecture) is completely ‘Astylar’, meaning unmixed, pure, and consistent ( Fergusson & Spiers, 1910). When comparing KT with other similar temples, Fergusson stated that it is significantly superior, particularly praising the grandeur of the edifice and its elaborate decoration, claiming that it is the finest in the world.

In Hindu mythology, the Sun God is believed to travel across the sky in a chariot drawn by seven horses, with his charioteer Aruna guiding the way ( Basham & Rizvi, 1956; Bedbak, 1999; Rao et al., 2015). In the parapets of the porch *,* rise the dynamic standing figures of celestial musician-girls playing flutes, drums, strings and cymbals to accompany the chariot for its daily course. Along with myriad dancing girls in the form of Odissi dance, anterior to the seven horses carved bas-relief in the wall of *nata mandapa* (dance hall) to chronicle the journey of the Sun God. This amusing architecture is a larger scale of European baroque art of the late 16 ^th^ century which has been mentioned by Fabri Charles Louis, Hungarian Archaeologist and Historian ( Fabri, 1974). With original thinking in design, the architectural design was to appease the Sun God ( Figure 3).

### Dimensions of the temple

**Figure 4.**
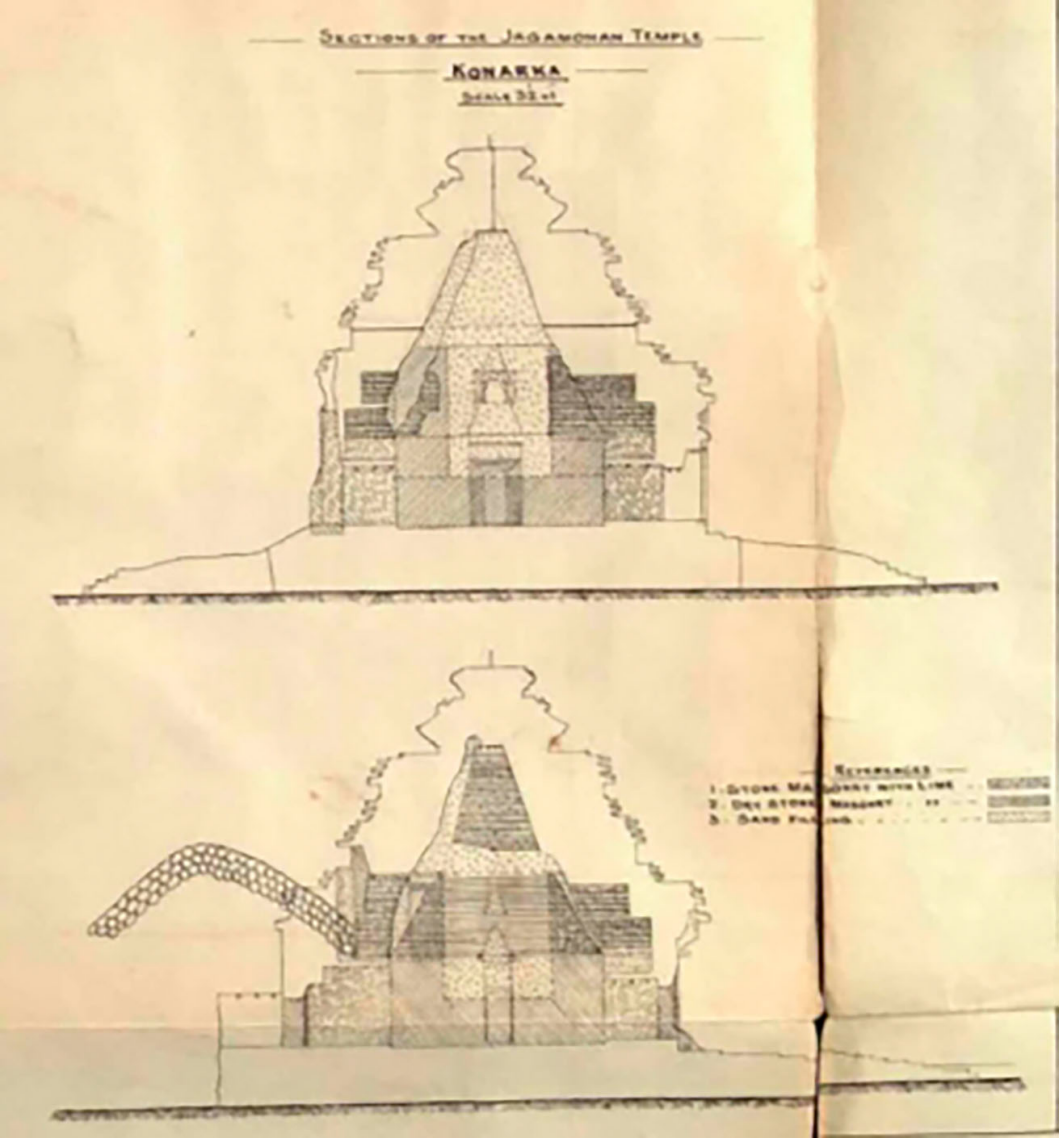
The interior layers of the porch ( Swarup, 1910).

The KT is thought to have consisted of a main *vimana* (sanctum), a *jagamohana*, a *nata mandapa* that is currently lacking in its superstructure, and a *bhogamandapa* (offering hall) that is in a state of ruin. Adjacent to these constructions, there were additional minor temples devoted to various deities, including the Aruna pillar which was relocated to Puri during the Maratha period and placed in front of *simhadvara* (main gate) of *Jagannath* temple ( Behera, 2005; Boner *et al.*, 1972; Dey, 2016; Donaldson, 2003; Mahapatra, 1989; O’Malley, 1908; Stirling, 1825; Sterling, 1846; Swarup, 1910; Tripathy & Kulke, 1987). The porch has been relatively well-preserved, but it was filled with sand in 1903 on the directive of Sir John Woodburn, the Lieutenant Governor of Bengal. This measure was necessary at the time to protect the monument ( Donaldson, 2003). The deadening process of filling the sand to preserve the *Jagamohana,* was completed early in 1905 ( Swarup, 1910). Indeed, the porch is refilled using stone masonry with lime, dry stone masonry with lime and sand, layered in various compositions ( Figure 4). The current porch lacks the main temple, as it has fallen due to multiple reckless depredations. The underlying mode of this monument is a colossal chariot adorned with twelve pairs of intricately carved wheels, designed for the Sun God.

### KT’s measurement

**Figure 5.**
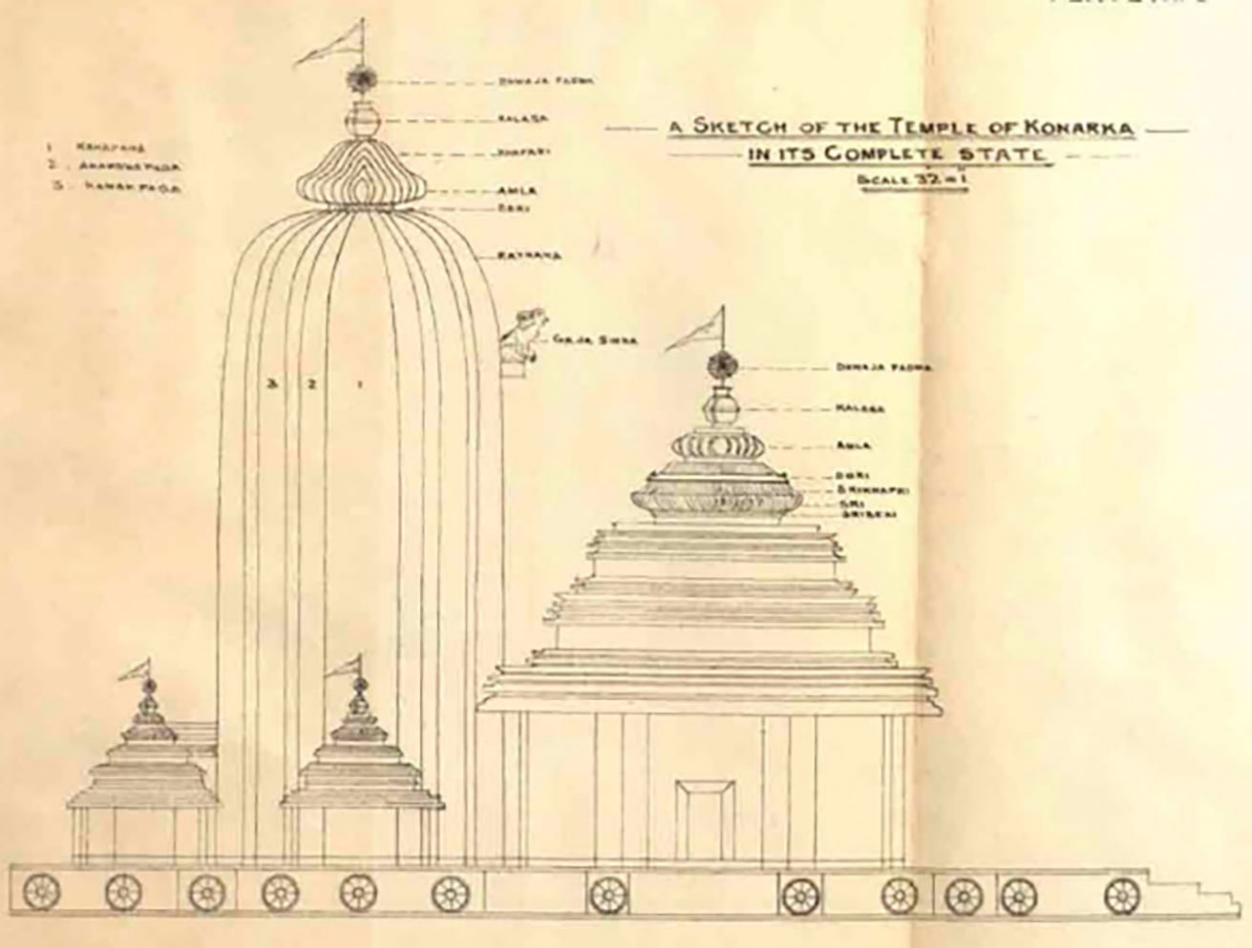
Sketch of the KT in its complete state ( Swarup, 1910).

The first measurement of the KT was made by Gajapati Purusottama Deva during his 4 ^th^ *anka* in the year 1610 A. D ( Dey, 2016; Donaldson, 2003), in which the height of the sanctum estimated was 68.5m (224.73 ft). Again, the measurement was undertaken in 1627 A. D, in the presence of Gajapati Narasimhadeva II, who was the son of Purusottama Deva and the grandson of Ramachandra Deva. The task was carried out by Natha Mahapatra, under the supervision of Bakhar Khan, who served as the subedar of Orissa under the Mughal emperor Jahangir ( Chakravarti, 1908). The Sanskrit version of the *Madalapanji,* has been acknowledged as an authentic account ( Behera, 2005; Ganguly, 1912; Mishra, 1919; Swarup, 1910). Translating the Sanskrit version of the *Madalapanji,* it was observed that Gajapati Narasimhadeva II inspected the vacant temple and recorded its dimensions. The temple complex was measured using a native method that took into account the finger width of the king. A unit called *kathi* (stick) was established, with 28 *angula* (finger width) being equivalent to one *kathi.* Local calculations have been revised to correspond with modern measuring standards, accounting for the width of an adult’s finger, with one *kathi* equating to 19 in. To corroborate the local calculations, the height of the porch had been considered, the only surviving segment of the monument, for which the height is known. During the excavation, Swarup (1910) meticulously measured almost every part of the temple complex, focusing especially on the height of the porch; the height of the porch from the platform level was recorded as 112 ft and 5 in, excluding the *Kalasa* (finial) and *Dhwaja* (flag), which were absent, instead, a piece of iron still protruded from the center of *Amala* (crown stone) ( Figure 4), supporting both. According to measurements, the platform height of the Temple from the plinth is recorded as 13 ft and 3 in. ( Ganguly, 1912; Swarup, 1910). Consequently, the height of the porch above ground level is calculated to be 125 ft and 8 in, not including *Kalasa* and *Dhwaja.* Ganguly (1912) calculated the height of the porch above ground level utilizing theodolite which was found to be 129 ft and 8 in. Referring to measurements taken during 1627 A. D, the height of the porch was recorded as 73 *kathi.* Converting this to feet by multiplying by 19 in, it was found to be 1387 in, which equals 115 ft and 7 in. Moreover, with the inclusion of the height of the platform, which is 13 ft and 3 in, the total becomes 128 ft and 10 in. This closely aligns with the calculation of Ganguly, deviating by 3 ft and 2 in from Swarup’s measurement due to the omission of the height of *Kalasa* and *Dhwaja.* Including the height of the porch, the measurement taken in 1627 A. D was factual. The measurement of the sanctum was conducted precisely, considering various segments. For instance, the measurement from below the lion upon *Amala* to the platform is 87 *kathi*, with an additional 12 *kathi* and another 12 *kathi* from the lion to *Garuda*, totaling 111 *kathi* which equals 175 ft and 9 in. Similarly, the measurement from above the lion of the *Amala* is recorded as 21 *kathi* 3 *angula*, equivalent to 33 ft and 5 in, excluding the *Kalasa* and *Dhwaja*, which were absent during the measurement. Thus, the total height of the sanctum from the platform is 209 ft and 2 in without *Kalasa* and *Dhwaja.* According to the measurements specified in the *Madalapanji*, the combined length of the *Kalasa* and *Dhwaja* is recorded as 3 *kathi* 8 *angula*, which is equivalent to 5 ft and 3 in. Including the height of the *Kalasa*, *Dhwaja*, and the platform from the plinth, which is 13 ft and 3 in, the total height of the sanctum reaches 227 ft and 8 in. This matches with Ganguly’s calculation of 228 ft ( Ganguly, 1912; Behera, 2005; Dey, 2016). Accordingly, the measurement taken in 1627 A. D was accurate, and the KT was the tallest temple in Odisha ( Figure 5).

During the measurement of the temple, the stone *kalasa*, adorned with a lotus finial, was absent from the sanctum, similar to the porch’s state when Swarup undertook the measurement. However, the iron rod mentioned by Swarup, which was positioned above the porch and referred to in the *Madalapanji* as “ *Chumbaka luha dharana*” (the magnetic iron rod), remained in place and projected above the top of the sanctum.

### Use of magnet

Based on the interaction with the nearby villagers, Sterling (1846) mentioned about a massive lodestone, which was believed to possess magnetic power capable of disturbing the direction of ships navigating through this route. Allegedly, a group of discontented Muslim sailors (Mughals) from troubled ships removed the magnetic stone ( Behera, 2005; Donaldson, 2003; Hunter, 1872; Jana *et al.*, 2021; Laurie, 2000; Lowe, 2016; Rath, 2021; Sterling, 1846), akin to the tale of ‘Sinbad the Sailor’s Rock,’ in hopes of resolving the navigation issues during their voyage. Forthwith, the priests migrated to the shrine in Puri. While a more precise conclusion remains contingent on affirmative evidence, an alternate rendition of the legend suggests that the image of the Sun God was suspended in the air through the use of magnets. Considering the memory of people, another legend revolves around the loadstone and the *Kalasa* atop the sanctum. It recounts the story of a person who completed the sanctum by installing the *Kalasa.* The story also speaks about the skilled manpower who constructed the temple.

### Recollections of construction and human resources

Given the absence of authentic records detailing the construction of the KT ( Dey, 2016; Jana *et al.*, 2021), the approach was to explore incidents recounted and heard from earlier generations by the local people. Among enigmatic legends encircling the construction of the KT, the most prevalent tale is of ‘Dharama’ or what localities recall as Dharmapada. He was the son of Bishu Maharana ( Jana *et al.*, 2021; Rath *et al.*, 2015), the chief craftsman in charge of the construction of the temple. Legend has it that, over twelve years, 1,200 carpenters and masons worked on constructing the temple. Before construction began, Bishu Maharana, the master craftsman, departed for the site as per the King’s orders, leaving behind his pregnant wife. Days later, she gave birth to a son named Dharmapada. After twelve years, Dharmapada learned that his father was constructing a great temple for the king, sparking his interest in meeting him. He sought permission from his mother to embark on a journey for this purpose. His mother then gave him the fruit of *Barakoli* (Apple Ber) from the tree in their courtyard as a symbol of recognition. After a long journey, he arrived in Konark and began selling fruits near the construction site. Bishu Maharana recognized the fruit from his courtyard and assured the boy that he was none other than his son. However, the joy of their union was short-lived as Dharmapada learned of a decree issued by the king. If the craftsmen failed to complete the sanctum up to the *Kalasa* by that night, the 1,200 craftsmen would face execution following morning. Upon hearing this, that very night Dharmapada ascended the temple and, with his craftsmanship, completed the sanctum up to the ‘ *Kalasa*’. The following morning, witnessing Dharmapada’s achievement, the artisans feared the consequences to be beheaded. Aware of risking their jobs and lives, they turned to Bishu Maharana for guidance. Reluctantly, he chose to save his fellow craftsmen over Dharmapada. Upon hearing this, Dharmapada immediately climbed to the top of the sanctum and jumped into the sea, sacrificing his life to save the 1,200 craftsmen. This narrative underscores Dharmapada’s selfless act for the greater good, set against the backdrop of the temple’s proximity to the sea.

This folktale, initially documented in 1876 A. D in “Dhagamala” by Kapileshwar Vidyabhushan, presents the adage “ *Barasha badheire daya ki puare daya*” (whether responsible to the 1,200 artisans or responsible to the son). O’Malley (1908) collected and published the story in English, differing slightly from the popular folklore. He changed it, depicting Bishu Maharana sorrowfully accepting the preference for his fellow workers and climbing to the top where his son was still working, intending to hurl him down to the pavement below. Nationalist writers like Kripasindhu Mishra ( Mishra, 1919) and Nilakantha Das (1919) supported O’Malley’s version. Pandit Gopabandhu Das (1924) introduced the name “Dharmapada” in his poem, emphasizing the theme of self-sacrifice. During the era of nationalism, Pandit Gopabandhu Das, a frontrunner in the Odiya nationalist movement, slightly tweaked the tale of Dharmapada to motivate the Odia youth, establishing the legend of self-sacrifice for the greater good. Ashvini Kumar Ghosh’s play, performed by the Annapurna group in Cuttack during 1950 A. D, revived the folklore and popularized the characters of Dharmapada and Bishu Maharana ( Das, 2011; Dey, 2016). Ghosh also introduced the name “Bishu Maharana” for the first time in the play ( Das, 2011).

It is evident that Dharmapada (Dharma Mahapatra) was indeed a real person ( Boner, 1970; Boner et al., 1972; palm leaves: L7, L12, L13, LI 1-11, LIII 4, LIII 7). He was the son of Sadashiba Samantaray Mahapatra, also known as Shivai Samantaray ( Boner, 1970), who served as the *sutradhara.* The palm leaves also mention Dharma Mahapatra’s significant role in completing the *Kalasa*, the crown stone of the temple. Additionally, other prominent artisans involved in the construction of the KT were: Narayana Mahapatra (master sculptor), Gadadhara Mahapatra (chief executive architect), Viswanatha Mahapatra (sculptor specialized in royal scenes), and Ganga Mahapatra (head of the *silpis*) ( Baumer & Konishi, 2007; Boner et al., 1972; Boner, 1970).

This established historical fact serves as the foundation of these folklores that passed from one generation to another through words-of-mouth. The construction of the huge structure of the Konark in ancient times was a group endeavour of 1,200 artisans, assigned by the head of the kingdom, under the supervision of many master artisans. Threat to the artisans by the king was the means for the completion of the construction of the temple work and Dharmapada’s self-sacrifice was the action to protect the insult and life of artisans’ community in ancient times.

### Obstacles during the early stages of construction

An anecdote of note revolves around Shivai Samantaray, the master architect of the temple complex ( Mohanty, 2001; Rath *et al.*, 2015). The initial task entailed the placement of stones in a section of the Padmatola river gorge, which was a pool covered with lotus plants. The objective was to construct the temple in this filled area. Nevertheless, the stone blocks, that were being dropped into the water, were being carried away by the powerful currents of the gorge. Shivai Samantaray regularly beseeched the Goddess Ramachandi, whose temple was situated near the building site, to proceed in the construction site. Once, the deity Ramachandi manifested in the form of an elderly lady and presented him with a portion of steaming *Khiri* (a sweetened porridge), for consumption. At that juncture, he was both disturbed and famished. Without any hesitation, he began consuming the gruel from the centre of the dish, which caused his fingers to be burned and subsequently pulled back and recoiled. The disguised goddess chuckled at him and advised him to consume the meal from the periphery, unlike Shivai Samantaray who was attempting to fill the gorge with stones, that is, from the centre instead of one side of the gorge. This incident served as a revelation for Shivai Samantaray, enabling him to effectively accomplish his task of filling the canyon. Upon receiving the solution from the elderly woman, purportedly the deity Ramachandi, Shivai Samantaray initiated the creation of an island by extending a bank and filling the gorge, thereby establishing a stable foundation for the entire structure.

### Foundation of KT

**Figure 6.**
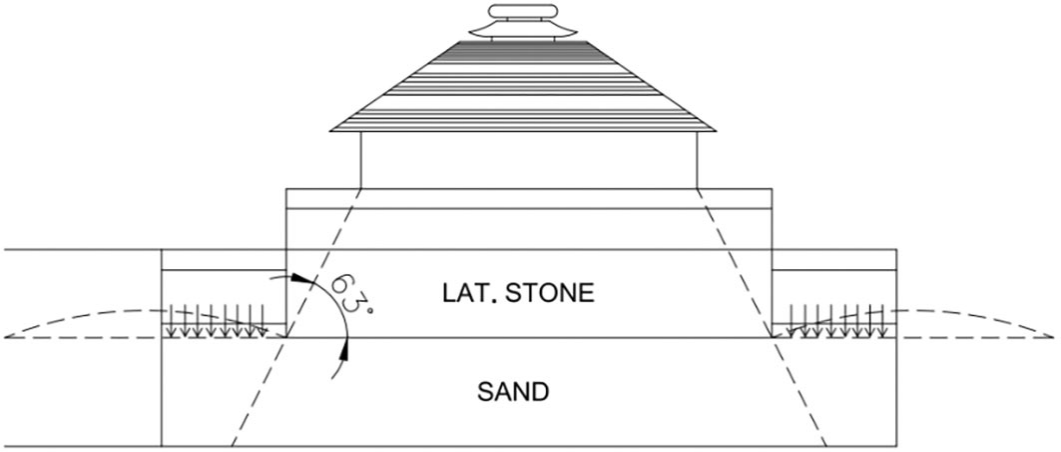
The engineering aspect of the foundation of KT (Source: Authors).

Based on a recent scientific investigation by the Central Building Research Institute (CBRI), Roorkee, the foundation of the KT consists of a block-type foundation that is 10.82 m deep ( Dwivedi *et al.*, 2022). The foundation comprises layers of Khandolite and Laterite stones, with the lowest layer being a dense sand stratum extending further for 11.5 m. In total, the foundation measures 22.32 m in depth (see Figure 6).

The most suitable foundation for an architectural plan in a sea beach location, according to modern engineering practice, is a block-type foundation with a sand footing over a compacted layer of dense sand. According to the norm, the foundation of a temple structure should typically be one-third of its height ( Meister, 1985; Kak, 2017). Previous records indicate that the height of the platform from the plinth of the KT was documented as 13 ft and 3 in ( Swarup, 1910; Ganguly, 1912). However, the current measurement in the cross-section indicates 4.12 m, equivalent to 13 ft and 6 in. Subtracting the platform height from the sanctum height, it amounts to 214 ft and 6 in. This comparison with the foundation, which measures 22.32 m (73 ft and 3 in), reveals a well-maintained height-to-foundation ratio, showcasing the remarkable technical expertise and skill of the *sthapati* (architect) of Kalinga. Geotechnical engineers’ research has established that the rate of consolidation of sand is higher than that of silt, clay, and other soils ( Das & Sobhan, 1990), making it a suitable material for filling below the foundation. Sand can also be compacted rapidly by saturating it with water, thereby reducing the need for the application of compaction energy. The thickness and width of the khandolite stone and laterite stone layers below the platform level were carefully determined to achieve a load dispersion angle close to 60°, which is less than the dispersion angle observed with stones ( Figure 6). Due to the high dead load of the 10m thick stone platforms and the temple itself, there is a risk of general shear failure and soil heaving. To mitigate this risk, artisans have incorporated 5.5m stone layers around the main stone foundations, demonstrating their sound engineering knowledge in the construction of the KT (see Figure 6).

### Stone selection in KT

The architectural framework of the temple and the sculptures were constructed using Khondalite stones, supplemented by Laterite and Chlorite ( Dey, 2016; Donaldson, 2003; Nayak *et al.*, 2017; Saxena & Srivastava, 2021). Laterite stones were mostly utilised in the subterranean section, situated beneath the plinth level, for foundational purposes; Chlorite stones were specifically employed for ornamental purposes, such as crafting statues of the Sun God, the *Simghasana* (pedestal), the puja image, *Nabagraha* (nine planet) statues positioned above the lintels, the *Aruna stamba* (Aruna pillar), sculptures housed within mundi niches, and other decorative elements found in the doorframes. The meticulous selection of stones was subjected to remarkably minimal decay over time, compared to other renowned structures such as St. Paul’s Cathedral and Norwich Cathedral. St. Paul’s Cathedral eroded by 30 mm in 250 years, and 95% of the outer stones of Norwich Cathedral were renewed during nine centuries, whereas the decay of the KT was only 2.4 mm ( Behera, 2005). In an investigation conducted by UNESCO ( Lemaire & Laurenzi Tabasso, 1981), the condition of the stones was found to be satisfactory following chemical testing of the existing materials. Despite the close proximity of the temple to the sea, the stones have not suffered significant damage, indicating the remarkable foresight of the people who selected stones, even in the 13th century.

### Stone quarrying and transportation of stones

**Figure 7.**
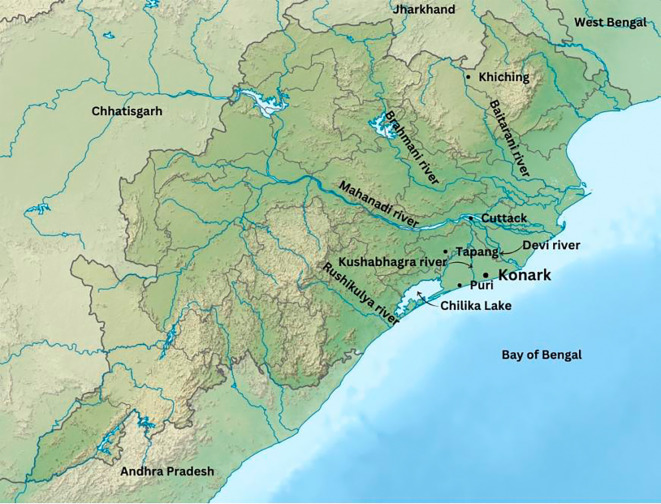
The river system connected to the KT site (Source: Authors).

Upon examining the outskirts of Konark, it is evident that there are no stone quarries in close proximity, even within a range of 30-40 kilometers. Stones were obtained from various locations including Naraj, Narasinghapur, Siddha Durga, Jagadalpur, Tapang, Ghantasila, Neelagiri, and Khiching, from a distance of 40 to 290 km from the temple structure ( Behera, 2005; Boner *et al*., 1972; Donaldson, 2003; Dey, 2016). When the temple was constructed, there were no efficient means of road transportation, and people did not utilise mechanised vehicles. People who visited the temple from the late eighteenth century to the early twentieth century noted that there was a lack of appropriate road communication ( Boner *et al.*, 1972; Cumberland, 1865; Fergusson, 1848; Hunter, 1872; Kittoe, 1838; Mishra, 1919; Mitra, 1880; Stirling, 1825; Sutton, 1833; Swarup, 1910). According to James Fergusson, waterways were utilised to transport stones that were subsequently looted from the KT and Barabati Fort in order to construct a lighthouse at False Point by the Europeans ( Fergusson, 1848; Fergusson & Spiers, 1910). Consensus prevails that water served as the mode of transportation for conveying the stones to the site ( Behera, 2005; Boner *et al.*, 1972; Dey, 2016; Donaldson, 2003; Fergusson, 1848; Fergusson & Spiers, 1910; Jana *et al.*, 2022; Mishra, 1919; Saxena, & Srivastava, 2021). Upon analysing the origins of the stones and the water connectivity, it is evident that, except Naraj, Narasinghapur, and Siddha Durga, there are no direct river connections to the work site. These three locations are in close proximity to the banks of the Mahanadi river. The Mahanadi river served as the lifeline of the region, intricately connected with other rivers. This was a key consideration in the Eastern Ganga dynasty’s choice to move their capital from Kalingapatnam to present-day city of Cuttack ( Banerji, 1930; Chinnappa, 1978; Panigrahi, 1986; Rao, 1941; Singh, 1973; Sundaram, 1963). It enabled smoother travel along the Mahanadi river and its tributaries, enhancing communication and connectivity with other kingdoms. Additionally, the geographical position seemed to be central, facilitating interactions from both the north and south of the kingdom. In the era of the Eastern Ganga empire, the capital resided at the convergence of the Mahanadi river, precisely where it bifurcates into several branches, closer to the Barabati Fort cum Palace. One of the tributaries of Mahanadi, the Kushabhadra river, flows into the Bay of Bengal near the KT ( Jana *et al.*, 2018). Examining alternative stone quarries and transportation methods, the construction site presents a challenge to transport heavy materials upstream of rivers, such as from Jagadalpur, Tapang, and Ghantasila in the Chilika lake zone ( Figure 7). The proposed route involves travelling upstream of the Daya river and then connecting with the Kushabhadra river downstream to reach the site. Nilagiri does not have a direct river connection ( Acharya, 1955; Dey, 2016; Donaldson, 2003). However, there used to be a water path called *Bhirudi Nala* (Bhirudi Canal) that connected Nilagiri to the Salandi river downstream ( Dey, 2016). From there, it was possibly flowing through the downstream of the Baitarani river and connected with the Birupa river, a distributary of the Mahanadi river upstream, eventually reaching Cuttack. To reach the site, one would then need to follow the course of the Kushabhadra river. Another location, Khiching, is easily accessible the downstream of the Baitarani river, and then the upstream of the connecting Birupa river, following a similar route as transportation from Nilagiri (see Figure 7).

### Logistics of stone transportation

During the time of 1870 A.D., according to the estimation and firsthand account of Hunter (1872), the rivers had the ability to transport weight, taking into account their depth and width. Specifically, the river routes that were used to transport stones and other materials to the construction site of the KT could carry a capacity of 20-25 tonnes ( Hunter, 1872), sometimes even more, particularly during the rainy season. There were lot many huge sculptures and stone blocks used in the temple which were more than 25 tonnes, one example of a large stone block is the monolithic *Nabagraha* slab weighed about 28 tonnes (Indian Museum, 1893), was originally over the lintel of the eastern door of the porch. As per eyewitness experience of Sterling, during his visit in 1822 A.D., that he had made a drawing of it when the *Nabagraha* slab was at its position, he compared it with that of the Gothic art of European architectural ornamentation, even in this dilapidated state looking at chlorite stone works around the doorways he said that the sculptures seem as if these came out from the chisel of the sculptors ( Sterling, 1846). Even, when Fergusson visited in 1837 A.D., it was at its position ( Figure 3). Observing the *Nabagraha* slab missing from its original position, Ferguson said when he visited again around 1869 that “an abortive attempt was made to carry the lintel to Calcutta”. During British rule over India, an attempt had been made by the Asiatic Society to carry the *Nabagraha* slab to the Indian museum, Calcutta, on 11 ^th^ January 1869 A.D., but failed due to its heavy weight and low fund allocation for the execution of the job (Indian Museum, 1893). As per the same report of the Indian museum, a second attempt had been made in 1892 A.D. by slicing the large piece longitudinally into two considering the uncarved portion of the slab from its back to reduce the weight; but again, failed due to the opposition from natives ( Ganguly, 1912). Despite attempts to transport the slab via waterways, it proved unsuccessful, leading to the decision to transport it by road. In fact, the *Nabagraha* slab was observed loaded onto a truck in front of the temple ( Mitra, 1880). Moreover, the *Amala* of the Sanctum was measured by the Archaeological Survey of India, which excavated a hole through the stone block, revealing a measurement of 25 ft and an estimated weight of 2,000 tonnes ( ASI, 1906). Considering the logistic challenges posed by the river’s width and depth, it seems impossible to transport such a large stone via river routes; instead, sea routes must have been utilized for this purpose.

### Maritime legacy: Navigating ancient sea routes and seafaring knowledge

The tradition of river and sea routes is deeply rooted in Odisha’s history, reflecting its cultural heritage and maritime past. Festivals like *Boita Bandana* (ritual of boat worship) during *Kartika Purnima* (full moon day in October or November month) and *Khudurukuni Osha* (a traditional fasting ritual observed by unmarried girls in Odisha) honour this legacy, with symbolic boats set afloat to commemorate the state’s maritime heritage ( Das, 2020; Guy, 1999; Sahoo, 2017). Additionally, festivals like *Chaiti Ghoda* (a folk festival featuring small wooden horse in March or April) and *Brata Khulanasundari* (vow of a legend Khulanasundari) celebrate the connection to maritime activities, with fishermen worshiping their boats and tales of sea voyages intertwined into folk practices ( Behera, 1999; Das, 1991; Das, 2020). These festivals celebrated throughout the year, serve to preserve the memory of Odisha’s maritime history through folk traditions and tales. The interpretation of inscriptions found at Udayagiri and Khandagiri Caves in the post-Buddha period, it becomes apparent that ancient Kalinga was a prominent hub of trade and commerce ( Prinsep, 1837). Due to its advantageous geographical position, Kalinga’s monarchs once wielded significant influence throughout India. Young princes received education in *‘Nawa-Byapara *’ (ship-commerce), highlighting the longstanding tradition of maritime trade and commerce education among monarchs dating back two thousand years. When English merchants established their first factory in Odisha near Harishpur Garh in the Mahanadi delta in 1633 A.D. ( Wilson, 1895; Bowrey, 1993), under the leadership of Mr. Cartwright, they obtained permission from Mughal Emperor Shah Jahan at the state palace of “Malcandy” (Mughals’ renaming the Barabati Fort cum Palace as such) in Cuttack, Orissa. Harishpur Garh, located at the river mouth, held historical significance for sea trade, as indicated by the names “Boita-kuilya” and “ship-haven,” ( Bowrey, 1993; Wilson, 1895); reflecting the maritime traditions of the Kalinga kingdom that persist in present-day folk culture, in the festival of *Boita Bandana.*

The maritime activities of ancient Kalinga drew the attention of artists to be depicted in the form of sculptures at different places. An interesting sculpture preserved at Orissa State Museum depicts the journey of a boat over water carrying people along with elephants. A 12 ^th^-century sculpture of a reverse-clinker boat is portrayed in the *Bhogamandapa* of Jagannath temple; the block of sculpture with the splash of oars, and ripple of waves indicates the desperate speed of the boat ( Barnes & Parkin, 2015; Behera, 1999; Bhowmick, 2005; Mookerji, 1912; Nayak, 2009; Parkin, 2016); as if, it is escaping from a danger: purportedly it was brought from the KT. There are two other boat sculptures of the medieval period from ancient Orissa preserved in the museums of Victoria and Albert Museum, London, one of which is labelled as *Khelana* (toy) instead, the name of the boat is supposed to be Khulana as it is known for Bhowmick (2005). An illustration of a boat pettoo-a “from Balassora or the coast of Palmira” by a Flemish Marine painter Frans Balthazar Salvyns displayed in the National Maritime Museum Greenwich, which is the reminiscent of Patia boat of Orissa ( Behera, 1999; McGrail, 2001; McGrail *et al.*, 2003). The construction of the Patia boat is considered as one of the most complex traditional boats in the world ( Bhattacharrya, 2006; Blue *et al.*, 1997). Even a few more museums of India and abroad have evidence of different types of ancient boats of Kalinga preserved, which reveal ingenious skills. There were different types and sizes of boats used by the people that has been detailed in a Sanskrit book *Yukti Kalpataru* (wish-fulfilling tree) which elaborates on the ship building along with measurement and varieties 25 names of ships ( Mookerji, 1912; Sahai, 1996; Sastri, 1917; Sinha, 1999). There is a temple of the 8 ^th^ century named *Boitala Deula,* in Bhubaneswar, Odisha, the name of the temple suggests it is having the resemblance of a *Boita* (ship). The temple is also giving evidence of the sustained level of maritime activities.

Archeological survey reports that a port was discovered at Khalkatapatana near the banks of the river Kushabhadra ( Bandi, 2000; Manjhi *et al.*, 2000; Sinha, 1999), in close proximity to the KT. Excavation efforts led by K. Veerabhara Rao and his team led to the conclusion that an active port-town existed during the 12th to 15th centuries ( Bandi, 2000; Tripati *et al.*, 2015a, 2015b). However, the port-town declined following the downfall of the Eastern Ganga dynasty. This affirms that the sea routes and ships were used for transporting huge stone blocks during the construction of the Sun Temple.

### Stone elevation technology

Evidence emerged suggesting that ancient artisans possessed the expertise to move the massive stone blocks. An intriguing example is the weight of the crowning stone of 2000 tonnes, with a thickness of 25 ft. It was one of the largest stones in the world ( Childress, 2013). There are other monumental stone blocks arranged in the shape of sculptures at varying elevations, featuring impressive figures such as the huge *Gaja singha* (lion upon elephant), *Nabaghraha* slabs of different lintels, a lion trampling on an elephant and musicians positioned at different heights, and other large sculptures. Childress (2013) hypothesized that advanced technology was employed during the construction of the edifice, such as large-scale saws, power grinders, drills, and some unidentified method of levitating massive stones or rendering them weightless. Childress’s assumption that the ancient people made heavy stones weightless using their scientific knowledge. As explained in ‘Baya Chakada’, the heavy lifting was accomplished through the use of scaffolding, skilled workers, various tools, and most importantly, trained elephants. Records of this tradition are evident in a sculpture panel originally housed in the KT, now gracing the walls of the Siddha Mahavir temple in Puri. These sculptures, relocated to the Siddha Mahavir temple during the Maratha period ( Behera, 2005; Dey, 2016; Donaldson, 2003), vividly depict the method of hoisting massive stone blocks with the aid of scaffolding. In the scene, two individuals are seen working at the top while four masons carry a rectangular stone block along an inclined path. Three elongated pillars are visible, providing support to the inclined slope, with one end positioned on the temple surface and the other resting on the ground. In conclusion, scaffolding was constructed of wood ( Behera, 2005; Dey, 2016). This also refutes the notion of filling sand inside and outside the temple to aid in transporting stone blocks and other materials for smoother construction progress. However, considering the substantial use of iron beams and clamps throughout the temple by ancient builders, it is likely that they employed iron beams, rods, and clamps for the scaffolding used in lifting large stone blocks.

### Native technology

The use of enormous stone blocks and colossal iron beams in the construction is remarkable to all the scholars who have visited the site, ranging from Mahmud Bin Amir Wali, and Baba Brahmachari to European and Indian scholars of the 19th and 20th centuries, as well as those visiting today. To ensure the stability of the building, heavy stone blocks were strategically placed on top, which was the primary function of the *Amala* ( Swarup, 1910). The term ‘ *Amala*’ is abbreviated from "Amara-shila," indicating that the “sila” (stone) was intended to sustain the structure as ‘ *Amara*’ (immortal). Furthermore, another heavier stone block was selectively placed beneath the *Amala*, similar to the porch ( Figure 5), to make it more stable. This demonstrates that the builders were keenly aware of the importance of weight distribution while designing a large structure.

Considering iron beam were neither utilized for decorative purposes in the temple nor were employed in the construction of the *Vijayastamba* (triumph pillar), similar to the pillars in Delhi or Dhar. Every individual iron component utilized in the temple’s construction served the purpose of providing support to various sections and corners, as well as reinforcing the monument. In addition to natural adhesives, iron clamps were frequently employed in construction to strengthen the seams. As per Swarup’s measurements, the porch is a square hall, spanning 60 ft by 60 ft, upheld by four pillars and reinforced with robust iron beams to support the ceiling ( Swarup, 1910). Additionally, beams were integrated into the lintels to strengthen the structure and uphold the sizable *Nabagraha* slabs positioned above each doorway of the porch. Moreover, numerous beams were installed as a false ceiling to offer additional support, alongside the extensive use of iron clamps in the stone joints. During the course of demolition, nearly all of the beams were fragmented, with the longest pillar measured to be 35 ft ( Graves, 1912). Fergusson (1910) noted that the beams in the ceiling had variable thickness, gradually widening from the sides towards the centre. It is indicative that artisans had expertise in the technical aspects and had the ability to use knowledge about the strength and properties of the metals being utilized.

### Use of high-grade iron

The ancient artisans were able to shape and join large iron beams over eight centuries ago. Engineer M.H. Arnott, who was involved in the rehabilitation crew during the early 20th-century excavation, discovered the manufacturing secret when he examined a broken iron beam. The iron was forged into small pieces of one to one and a half feet in length and three to four inches in width. These pieces were then arranged side by side in a row, similar to bricks in a wall ( O’Malley, 1908). The segmented components were inserted into a quadrilateral hollow iron bar and then welded together to create a secure and seamless connection. The upper surface was subsequently polished to eliminate any visible signs of the joints, resulting in a robust and sturdy beam. The material’s composition is akin to contemporary military-grade steel ( Edwards, 1969; Friend, 1926; Singh & Kaur, 2014). Moreover, in order to obtain certainty, Newton conducted a test to directly compare the corrosion resistance of the iron of the KT with modern military-grade steel ( Friend, 1926). To do this, he obtained samples of both metals that were identical in shape, size, and weight. These samples were then exposed to various atmospheric conditions, including normal water wet and dry tests for one year, followed by an artificial sea water test for an additional year. Following a two-year scientific observation, he found that the resistance capacity of the iron used in the KT surpasses the contemporary military-grade steel. This finding was a learning lesson to him and to the Iron and Steel Industry. Such observation questions the correlation between technological advancement and the quality of products produced by the new generation, encapsulated in the adage “the good die young?” In order to test the limits of current technology, numerous lengthy iron beams have been left in place inside the KT for almost four centuries. The mixture of technical proficiency with local knowledge in ancient times provides a remarkable level of dexterity to the art of constructing buildings, which is impressive to the globe today.

### Astronomical wonder: Illuminating the magnificence of construction

The KT’s wheel functions as a vertical sundial, meticulously designed to align with the exact latitude of Konark ( Bhatnagar & Livingston, 2005; De, 2022; John, *et al.*, 2015; Joshi & Srivastava, 2021; Yadav, 2021). This design allows for the accurate determination of time throughout the day, with minimal error, by casting shadows when a long stick is positioned at a zero-degree angle to the axle of the wheel, parallel to the ground in relation to the angle of the sun. The circular wheel is divided into eight major spokes, each representing a three-hour interval, effectively dividing the twenty-four-hour day. Between each major spoke, there are eight minor spokes, further dividing the space into halves and indicating intervals of one and a half hours, or ninety minutes. Additionally, thirty beads are positioned along the edge of the wheel between a major and a minor spoke. Each bead corresponds to three minutes of time. Moreover, by observing the gap between each bead and its center, finer subdivisions of time are discernible, with intervals as precise as one and a half minutes. The sundial functions in a counterclockwise manner. This intricate design showcases the ancient sculptors’ knowledge of astronomy, making the wheel an instrument for celestial timekeeping.

The temple was built with precision, enabling the first ray of the rising sun to penetrate through the porch and illuminate the idol of the Sun God in the sanctum of the KT ( Das, 2015). A discussion with Soumit Biswal, a scientist from ISRO, the Vikram Sarabhai Space Centre, Thiruvananthapuram, Kerala, suggests that the temple is aligned along the east-west axis with one-degree accuracy. Observations indicate that on March 21st and September 22nd, twice a year, the sun’s rays were expected to directly illuminate the idol. Taking into account the width of the main door of the porch, Suvendu Patnaik of Pathani Samanta Planetarium, Bhubaneswar, suggests that sunlight would fall for approximately thirty days twice a year: from March 6 ^th^ to April 5 ^th^ and from September 7 ^th^ to October 6 ^th^ ( Dey, 2016). He further specifies that with an opening of one degree, the sun illuminates the area for about 11-12 days during each of these periods annually. This also validates the name of the monument, KT, derived from two Sanskrit words *Kona* meaning angle and *Arka* meaning Sun. It signifies the precise *Kona (*angle) through which the rays of *Arka* (sun) enter to illuminate the idol of the Sun God in the temple.

## Discussions

### Summary of findings

The study sheds light on varied aspects of the KT construction. It uncovers the purpose behind the temple’s construction, emphasizing its cultural and religious significance within the kingdom’s context. Additionally, the relative security of the kingdom during construction implies a stable political environment, revealing the king’s achievements in different wars and contributing to monumental projects. The identification of diverse artisans involved underscores the collaborative effort and diverse skills required for such endeavors. Trained elephants played a vital role in providing support during the construction process. Additionally, they were employed as a formidable troop in the wars waged by King Narasimhadeva I, earning him the title of Gajapati, which he proudly bore as the first king of Kalinga.

The temple sculptures act as repositories of encrypted facts and historical narratives, enriching the understanding of that era. The accuracy of the temple’s measurements, as recorded in the Sanskrit version of *Madalapanji* dating back to 1627 A.D., has been confirmed through translation. The meticulous selection of high-quality stones demonstrates dedication to craftsmanship. Innovative transportation methods like sea routes and ships, alongside the use of iron scaffolding, highlight advanced engineering techniques. Notably, lifting the world’s heaviest stone to a height of around 200 ft showcases remarkable engineering prowess. The knowledge of utilizing iron ores for rust-resistant iron exemplifies the ingenuity of ancient craftsmen. Again, the intricately crafted wheel of the KT serves as a vertical sundial, perfectly aligned with the precise latitude of Konark. Its construction allows the first ray of the rising sun to illuminate the idol of the Sun God within the sanctum, showcasing ancient sculptors’ mastery of astronomy and architectural precision.

### Implications

The advanced indigenous technology utilized in the construction of the ancient KT showcases remarkable sophistication, demonstrating a fusion of artisans’ skills, commitment, folk knowledge, and the quality of construction materials. Delving into the intricacies of this indigenous technology illuminates historical architectural achievements and revitalizes the forgotten art form of Kalinga, offering insights into ancient construction techniques.

### Limitations

The limited journal publications on KT restricts the access to authentic evidence. Nevertheless, the archival data are employed from indigenous knowledge dating back to the 13th century, and folk stories and prevailing cultural practices. Documentation includes incidents and eyewitness accounts found in books, diaries, palm leaf inscriptions, and stone inscriptions. Furthermore, the study involved analyzing sculptures from the KT, along with those housed in museums of different locations, and exploring the ancient maritime traditions of the kingdom’s predecessors. Here the text also includes literature authored by King Narasimhadeva I’s court poet and historical records of the kingdom.

### Directions for research

The evidence surrounding the KT can be researched through multiple lenses. First, there are many festivals, cultural practices, and narratives that exist among the localities surrounding the KT as a reminiscence of the past. Those have passed on from one generation to another through observation of festivals, cultural practices, and words-of-mouth. Such knowledge of Dharma is derived here from secondary sources. Acquiring such folk evidence on festivals, cultural practices, and stories from primary sources through qualitative research and analyzing their contents and themes can provide insight into the history, cultural-orientation, craftmanship, and the science and technology surrounding the KT and its present state of affairs.

To further understand the construction of KT and shed light on the transportation of its massive stones, a geotechnical investigation can be carried out to analyze soil properties and borehole assessments to unveil ground conditions that may have influenced construction techniques and structural stability. Additionally, employing geophysical methods such as ground-penetrating radar (GPR) and electrical resistivity imaging (ERI) can help detect subsurface features like buried water channels or geological anomalies, while radar satellite data analysis can offer a broader perspective of the landscape, revealing hidden watercourses or geological formations relevant to the temple’s construction. Plus, Geographic Information System (GIS) technology facilitates the integration and analysis of various spatial data layers, including topography, hydrology, and historical maps, aiding in the identification of potential water bodies, ancient river courses, or coastal features that could have influenced the temple’s location and construction. Supplementing these methods with map analysis, including examination of historical maps, archaeological surveys, and aerial photographs, can provide valuable context for understanding the KT’s surroundings and changes in the landscape over time. By synthesizing these diverse investigative approaches, the research can provide a comprehensive understanding of the environmental context in which KT was built, unraveling its construction logistics, resource utilization, and the cultural and environmental factors shaping its design and placement in the landscape.

## Conclusion

This study has emphasized the significant contributions of an array of artisans involved in the construction process of the ancient monument of the KT. By exploring the true incidents preserved in the form of folk stories, cultural practices, and sculptures, the understanding of historical narratives has been enriched. Additionally, delving into the techniques and principles behind the ancient structures provides insights to architects and engineers to innovate and adapt improvised methods for sustainable and resilient monument design. Through this process, historical, cultural, and scientific evidence has been debunked, bridging the gap between past and present technologies to rationalize architectural excellence in the future.

This research reveals the enduring legacy of Narasimhadeva I. The ancient measurements of the temple, translated from the Sanskrit version of the *Madalapanji*, align with modern-day calculations, serving as a testament to the completion of this architectural marvel. Furthermore, our investigation unveils the remarkable utilization of sea routes for transporting massive stones to the construction site, shedding light on the logistical prowess of ancient engineers. Amidst the stone engravings, a poignant tale emerges, immortalized in sculptures, revealing the valor of Sudehi, the faithful elephant who played a pivotal role in reviving Narasimhadeva I from the battlefield. The artisans’ skills, knowledge, local wisdom during the 13th century played significant roles in the construction of the temple. The temple’s foundation has undergone modern engineering scrutiny and has been found suitable for an architectural blueprint in a seaside setting. It further unveils the feat of lifting the world’s heaviest stone to towering heights, a testament to the ingenuity of ancient construction techniques. As we delve deeper, we discover echoes of modernity within the ancient framework, with the utilization of iron scaffolding mirroring contemporary construction practices. Lastly, the inquiry unveils the enduring presence of rust-resistant iron within the temple compound. Alongside the temple’s wheel serving as India’s earliest sundial, offering a glimpse into the celestial intricacies revered by ancient astronomers. The first ray falling on the idol inside the temple is another astronomical keenness of ancient artisans. In this study, we find not just answers, but a gateway to further research on the KT.

## Ethical approval

Ethical approval and consent were not required

## Data Availability

No data are associated with this article.
