# Supplementary material for: The Konark Temple’s Construction: A Critical Review of the Historical, Cultural, and Scientific Evidence
Source: F1000Res. 2025 May 15;13:1540. Originally published 2024 Dec 20. [Version 2] doi: 10.12688/f1000research.157831.2 (PMC12203308; doi:10.12688/f1000research.157831.2)
Supplement: Peer review history

# Reviewer report: Reviewer response for version 2

**DOI:** 10.5256/f1000research.181512.r385522

**Published:** 2025-06-23

**Authors:** Isa Bala Muhammad

**Affiliations:** 1 Federal University of Technology, Minna, Nigeria

This study highlights the Konark Temple as a symbol of ancient India’s cultural, political, and technological achievements. It reveals the temple’s religious significance, the king’s military success, and the collaborative efforts of skilled artisans. Advanced engineering, astronomical precision, and rust-resistant iron showcase scientific ingenuity, while sculptures preserve historical narratives. Overall, the temple stands as a testament to the era’s architectural brilliance and multifaceted excellence.

Is the review written in accessible language?

Yes

Are all factual statements correct and adequately supported by citations?

Yes

Are the conclusions drawn appropriate in the context of the current research literature?

Yes

Is the topic of the review discussed comprehensively in the context of the current literature?

Yes

Reviewer Expertise:

Cultural landscape

I confirm that I have read this submission and believe that I have an appropriate level of expertise to confirm that it is of an acceptable scientific standard.

# Reviewer report: Reviewer response for version 1

**DOI:** 10.5256/f1000research.173339.r356835

**Published:** 2025-02-05

**Authors:** Sabeeh Lafta Farhan

**Affiliations:** 1 Wasit university, Kut, Iraq

Thoroughness & Scope – The article provides an extensive review of historical, cultural, and scientific aspects of the Konark Temple’s construction. The depth of research and integration of multiple sources, including historical texts, archaeological findings, and indigenous knowledge, is commendable.

 Interdisciplinary Approach – The combination of historical analysis, engineering insights, and cultural narratives enriches the discussion. However, a more structured comparison with contemporary temple architecture across different regions of India could provide additional context.

 Scientific and Engineering Aspects – The discussion on iron usage and rust-resistant techniques is fascinating. It would be helpful to include a comparison with other ancient iron structures, such as the Iron Pillar of Delhi, to establish technological linkages.

**Specific Comments:**

**Historical Narratives & Myths** – While the study effectively explores historical accounts and folklore, some claims (such as the floating deity using magnets) require additional corroboration from scientific sources or metallurgical studies.

**Methodology Clarification **– The systematic review method is well described, but the inclusion criteria for selecting historical texts, palm leaf manuscripts, and indigenous narratives should be elaborated. How were these sources validated for accuracy?

**Illustrations & Figures** – The inclusion of figures is helpful in visualizing the temple’s architectural layout. However, some of them lack detailed captions or references to their sources (e.g., Figure 5’s sketch of the temple in its complete state).

**Construction Techniques & Materials **– The identification of Khondalite, Laterite, and Chlorite stones is insightful. However, the decay rate comparison with Western structures like St. Paul’s Cathedral might need more quantitative backing with geological studies.

**Maritime & Transportation Hypothesis **– The theory of stone transportation via sea routes is intriguing. Have geological or sedimentary studies been conducted along these proposed routes to confirm ancient maritime trade or transport links? 

**References:** Lafta Farhan S.et.al. 2022 (Ref 1) This study discusses the transformation of historical city centers and could provide a comparative perspective on the cultural and architectural sustainability of heritage sites, similar to the preservation efforts and architectural significance discussed in the Konark Temple study. 

Is the review written in accessible language?

Yes

Are all factual statements correct and adequately supported by citations?

Yes

Are the conclusions drawn appropriate in the context of the current research literature?

Yes

Is the topic of the review discussed comprehensively in the context of the current literature?

Yes

Reviewer Expertise:

NA

I confirm that I have read this submission and believe that I have an appropriate level of expertise to confirm that it is of an acceptable scientific standard.

## References

[1] : The social transformation of the historical city centre of Karbala , Iraq. *Journal of Urban Regeneration and Renewal* .2022;15(3) : 10.69554/HJRS8205 10.69554/HJRS8205

# Response

**Published:** 2025-05-02

**Authors:** Susanta Bahinipati

**Affiliations:** Film and Media Sciences, Kalinga Institute of Industrial Technology, Bhubaneswar, Odisha, India

**Reviewer 2**

**Sabeeh Lafta Farhan**, Wasit university, Kut, Iraq 

 We are grateful for your appreciation of the work and constructive comments/ suggestions to improve the quality of the manuscript. 

**Thoroughness & Scope**

 The article provides an extensive review of historical, cultural, and scientific aspects of the Konark Temple’s construction. The depth of research and integration of multiple sources, including historical texts, archaeological findings, and indigenous knowledge, is commendable.

 Interdisciplinary Approach – The combination of historical analysis, engineering insights, and cultural narratives enriches the discussion. However, a more structured comparison with contemporary temple architecture across different regions of India could provide additional context.

 Scientific and Engineering Aspects – The discussion on iron usage and rust-resistant techniques is fascinating. It would be helpful to include a comparison with other ancient iron structures, such as the Iron Pillar of Delhi, to establish technological linkages.

 Thank you for your valuable feedback. We appreciate your recognition of the interdisciplinary approach and the depth of research. In response to your suggestion, we have incorporated a structured comparison with contemporary ancient iron structures, including the Iron Pillar of Delhi, in the revised section “Use of High-Grade Iron”.

**Listed Reply to Comments/ Suggestions**

**Historical Narratives & Myths**

 While the study effectively explores historical accounts and folklore, some claims (such as the floating deity using magnets) require additional corroboration from scientific sources or metallurgical studies.

**Response: **We acknowledge the need for scientific validation. Cultural accounts are presented, and future metallurgical studies are outlined in the third paragraph of the “Directions for research” section.

 Metallurgical and archaeological studies can also test historical claims, such as the possible use of magnets in KT.

**Methodology Clarification**

**The systematic review method is well described, but the inclusion criteria for selecting historical texts, palm leaf manuscripts, and indigenous narratives should be elaborated. How were these sources validated for accuracy?**

**Response: **The inclusion criteria for historical texts, palm leaf manuscripts, and indigenous narratives were based on their relevance to nine thematic areas. Sources were validated through cross-referencing with academic literature and comparative analysis with archaeological findings. These details have been incorporated into the *‘Methodology’* section, which has been revised as follows:

**Methodology**

 A systematic review methodology was adopted for the search and selection of documents. Documents were sourced from Scopus, the largest scientific journal database, and Google Scholar, an inclusive, automated repository offering access to a vast range of published works (Bar-Ilan, 2008). Two search strings were employed: “KONARK” OR “SUN AND TEMPLE” within the title, abstract, and keyword fields. This search yielded 329 documents from Scopus and 912 from Google Scholar. After removing duplicates (n = 235), the remaining documents were screened on the basis of titles and/or abstracts for relevance to nine thematic areas: (1) causes of construction, (2) financial allocation, (3) dimensions of Konark Temple (KT), (4) human resources, (5) obstacles during construction, (6) foundation of KT, (7) stone selection, transportation and lifting, (8) native technology, and (9) astronomical phenomena. This process resulted in 80 documents, comprising 29 journal articles and 51 books, and excluded 926 documents. Following a full-text review, 69 documents, containing 29 journal articles and 40 books, were retained as relevant to the aforementioned themes, while 11 books were excluded by the first author. The inclusion and exclusion of documents were subsequently discussed among all three authors, and the exclusion was agreed upon.

 Limited programmatic research and publications in journals on the KT resulted in the retention of 29 journal articles. As KT is an ancient structure dating back to the 13th century, 36 documents related to its architecture, construction, water bodies, and associated folklore were selected from cross-referenced old books and ancient writings (n = 29), palm-leaf inscriptions and indigenous narratives on stones (n = 6), and copper plates (n = 1). Also, 13 journal articles were selected for a comparative analysis of KT with other monumental structures worldwide (Aveni, Gibbs, & Hartung, 1975; Evans et al., 2013; Farhan et al., 2022; Gaffney et al., 2018; Ghoneim et al., 2024; Sheisha et al., 2022; Uchida & Shimoda, 2013), for assessing the quality of stone and iron used (Basu, Orr, & Aktas, 2020; Church of England, 2019; Ghosh, 1963; Gilchrist, 2001; Phys.org, 2012; Vincent, 1993). An additional journal article was included to rationalise the database for selection and inclusion of documents for review (Bar-Ilan, 2008). These were initiated by the first author and agreed upon by the remaining two authors, with no documents removed. Of 130 documents, 119 documents were included for this review (see Fig. 2). The inter-rater reliability (Number of agreed documents/ Total number of documents) was 91.54%.

**Illustrations & Figures**

 The inclusion of figures is helpful in visualizing the temple’s architectural layout. However, some of them lack detailed captions or references to their sources (e.g., Figure 5’s sketch of the temple in its complete state).

****Response: **Thank you for your feedback. Figure 5 cites its source in the legend (Swarup, 1910). We have reviewed all figures to ensure clear captions and citations. Minor revisions were made: in Figure 1. We have changed "Theoretical and practical implications" to "Implications"; in Figure 2, we corrected it as per the methodology section; and in Figure 7, we corrected "Kusabhagra" to "Kusabhadra."**

**Construction Techniques & Materials**

 The identification of Khondalite, Laterite, and Chlorite stones is insightful. However, the decay rate comparison with Western structures like St. Paul’s Cathedral might need more quantitative backing with geological studies.

****Response:** The ‘ *Stone selection in KT’* section has been revised to include a quantitative comparison of decay rates with Western structures such as St. Paul’s Cathedral and Norwich Cathedral, supported by geological studies for analytical rigor. Corresponding references have been added. The changes are as follows:**

**Stone selection in KT**

 The architectural framework of the temple and its sculptures were constructed using Khandalite stones, supplemented by Laterite and Chlorite (Dey, 2016; Donaldson, 2003; Nayak et al., 2017; Saxena & Srivastava, 2021). Laterite stones were primarily used in the subterranean section beneath the plinth level for foundational purposes, while Chlorite stones for ornamental features, including statues of the Sun God, the *Simghasana* (pedestal), the puja image, *Nabagraha* (nine planet) statues above the lintels, the *Aruna Stamba* (Aruna pillar), sculptures within mundi niches, and decorative elements on the doorframes.

 The meticulous selection of stones has resulted in remarkably minimal decay over time compared to Western structures such as St. Paul’s Cathedral and Norwich Cathedral. St. Paul’s Cathedral, built with Portland limestone, has undergone significant weathering due to environmental exposure. Studies show that its decay rate varies between 130–220 µm per year, leading to an estimated erosion depth of 40.82 mm to 69.08 mm over 314 years since its completion in 1710 A.D. (Phys.org, 2012; Basu et al., 2020). Additionally, long-term measurements using lead plugs indicate surface recession rates of 0.066 mm to 0.081 mm per year over 262 years, with a total erosion depth of 17.3 mm to 21.2 mm (Vincent, 1993). The highest deterioration occurs on surfaces facing southwest due to increased exposure to pollutants and weathering effects.

 Similarly, Norwich Cathedral, constructed primarily of Caen limestone since 1096 A.D., has undergone extensive erosion over centuries. The outer layers of its stonework have been significantly renewed (Church of England, 2019), with approximately 95% of its exterior stones replaced over nine centuries due to weathering, fire damage, and pollution (Behera, 2005). Historical evidence unravels the environmental exposure and structural modifications of Cathedrals over time, emphasizing the preservation of limestone-based heritage structures (Gilchrist, 2001).

 In contrast, the Khandalite stones of the KT have eroded by only 2.4 mm over 750 years (Behera, 2005). Such lower decay rate suggests that the stone’s intrinsic properties, along with environmental factors and historical preservation efforts, have contributed to its longevity. The chemical testing of existing materials of KT during 1979-1984 confirmed the satisfactory condition of the temple stones (UNESCO: Lemaire & Labasso, 1981).  Despite the temple’s proximity to the sea, the stones have not suffered substantial damage, highlighting the advanced scientific knowledge of 13th-century artisans in selecting durable materials resistant to coastal weathering.

 Reference

- Basu, S., Orr, S. A., &Aktas, Y. D. (2020). A geological perspective on climate change and building stone deterioration in London: Implications for urban stone-built heritage research and management. *Atmosphere, 11*(8), 788. https://doi.org/10.3390/atmos11080788
- Church of England. (2019). *Norwich Cathedral.* https://www.churchofengland.org/sites/default/files/2019-02/norwich-cathedral.pdf
- Gilchrist, R. (2001). Norwich Cathedral tower and spire: Recording and analysis of a cathedral's *longue durée.Archaeological Journal, 158*(1), 291–324. https://doi.org/10.1080/00665983.2001.11078997
- Phys.org. (2012, October 26). *Erosion of iconic St. Paul’s could benefit from laser scanning technology.* https://phys.org/news/2012-10-erosion-iconic-st-paul-benefit.html
- Vincent, K. (1993). *Atmospheric particulate matter and historic buildings* (Doctoral dissertation, Middlesex University).

**Maritime & Transportation Hypothesis**

 The theory of stone transportation via sea routes is intriguing. Have geological or sedimentary studies been conducted along these proposed routes to confirm ancient maritime trade or transport links? 

**Response: **Historical texts and the discovery of an ancient port at Khalkatapatana port suggest sea transport, but geological validation is needed. This is addressed in the third paragraph of the "Directions for research" section.

 Additionally, sediment coring, sonar, and bathymetric surveys using tools like sub-bottom profilers and remotely operated vehicles (ROVs) can explore potential maritime transport links.

**References**

 Lafta Farhan S. et.al. 2022 (Ref 1) This study discusses the transformation of historical city centers and could provide a comparative perspective on the cultural and architectural sustainability of heritage sites, similar to the preservation efforts and architectural significance discussed in the Konark Temple study. 

**Response: **Thank you for the suggestion. Lafta Farhan S. et al. (2022) is acknowledged in the fifth paragraph of the “Directions for Research” section to support future comparative heritage studies.

 Lastly, the transformation and sustainability strategies of historic city centers (Farhan *et al*., 2022), offer insights for preservation of KT. Tools like GIS-based urban analysis, 3D documentation, and heritage assessments, combined with adaptive reuse and conservation models, can support resilient, sustainable preservation.

**Reference:**

 Farhan, S. L., Alobaydi, D., Anton, D., & Nasar, Z. (2022). Analysing the master plan development and urban heritage of Najaf City in Iraq. *Journal of Cultural Heritage Management and Sustainable Development*.

# Reviewer report: Reviewer response for version 1

**DOI:** 10.5256/f1000research.173339.r356838

**Published:** 2025-01-09

**Authors:** Ngakan Ketut Acwin Dwijendra

**Affiliations:** 1 Udayana University, Denpasar, Indonesia

**The Konark Temple’s Construction: A Critical Review of the Historical, Cultural, and Scientific Evidence**

**Summary of the Article**

 The article critically reviews the historical, cultural, and scientific evidence surrounding the construction of the Konark Temple in Odisha, India. The research incorporates data from ancient texts, folklore, palm leaf manuscripts, archaeological evidence, and historical records. It explores themes such as the temple's purpose, the techniques and materials used, the role of indigenous knowledge, and its architectural and astronomical precision.

 Key findings include:

- The temple served as a monument of devotion to the Sun God and a symbol of King Narasimhadeva I’s military victories.
- Advanced construction techniques included using rust-resistant iron, innovative stonelifting technologies, and precise architectural designs.
- The temple's alignment with astronomical phenomena demonstrates ancient artisans' mastery of astronomy.
- Indigenous technologies, including the use of high-grade iron and efficient transportation of massive stone blocks via sea routes, highlight the engineering ingenuity of the time.

The article concludes that the Konark Temple embodies a blend of artistic mastery, cultural significance, and technological innovation, offering insights for contemporary architecture and engineering.

**Evaluation and Recommendations**

**1. Comprehensiveness of the Topic in Current Literature**

**Evaluation:** **Partly**. The article extensively reviews the historical and cultural significance of the Konark Temple, presenting a rich narrative of its construction and legacy. However, its engagement with contemporary research methodologies in archaeology, materials science, and geotechnical studies is limited. While the authors mention some modern studies, such as those by CSIR and CBRI, these are not deeply integrated into the analysis. For example, insights from recent archaeological technologies like ground-penetrating radar or 3D modeling could enhance the study’s relevance.

**Recommendations: **Incorporate more recent geotechnical or archaeological studies to substantiate claims about the temple’s construction techniques and materials. Review and discuss contemporary literature on similar historical structures globally for comparative insights.

**2. Factual Accuracy and Citation Support**

**Evaluation:** **Partly**. While the article is well-cited and draws on historical sources, its reliance on folklore and secondary sources raises questions about the verifiability of some claims, such as the legend of Dharmapada or the use of magnets to suspend objects. Some claims, like the rust resistance of the iron used, require more robust validation through referenced scientific studies.

**Recommendations: **Ensure that all key assertions, especially those based on folklore or oral traditions, are marked as such and distinguished from evidence-based claims. Include peer-reviewed scientific studies to corroborate statements about materials, such as the selection processes for rust-resistant iron and stone.

**3. Accessibility of Language**

**Evaluation:** **Yes**. The language is clear, engaging, and accessible to academic and general audiences. The integration of technical and cultural narratives is particularly commendable.

**Recommendations: **Maintain this clarity while adding more technical terms and methodologies if engaging with geotechnical or engineering audiences.

**4. Appropriateness of Conclusions**

**Evaluation:** **Partly**. The conclusions are generally appropriate, synthesizing the findings effectively. However, they are somewhat limited by the scope of the evidence presented. For example, the findings could better integrate critiques or limitations of ancient techniques compared to modern methodologies.

**Recommendations: **Discuss how the findings contribute to or challenge current historical architecture and engineering knowledge. Acknowledge gaps in evidence and propose specific areas for future research.

**Specific Points to Address for Scientific Soundness**

1. **Integration with Current Research **Include recent studies or methods, such as geotechnical assessments or 3D modeling, to validate claims about construction techniques. Provide a comparative analysis with other ancient monumental structures to position the findings in a broader context.
2. **Strengthening Evidence:** Use scientific data to support claims about material properties, such as the corrosion resistance of iron used in the temple. Provide citations from peer-reviewed journals, especially for claims about astronomical alignment and structural engineering.
3. **Distinguishing Between Fact and Folklore:** differentiate between historical records, archaeological evidence, and folklore. For example, legends like the story of Dharmapada should be framed as cultural narratives unless supported by evidence.
4. **Clarification of Methodology **Elaborate on the systematic review process, specifying how documents were selected and analyzed. Discuss potential biases in the sources.
5. **Addressing Limitations:** Acknowledge the study's limitations, such as the reliance on secondary sources and the lack of direct archaeological validation for some claims.

**Conclusion**

 Overall, the article offers valuable insights into the construction of the Konark Temple and highlights its historical, cultural, and scientific significance. The authors should address the abovementioned limitations to make the article scientifically sound, particularly by integrating more recent research, strengthening evidence-based claims, and distinguishing folklore from verifiable facts. By doing so, the study can serve as a robust reference for both historical and scientific investigations into ancient construction techniques.

Is the review written in accessible language?

Yes

Are all factual statements correct and adequately supported by citations?

Partly

Are the conclusions drawn appropriate in the context of the current research literature?

Partly

Is the topic of the review discussed comprehensively in the context of the current literature?

Partly

Reviewer Expertise:

Architecture, engineering, sustainable development, and cultural heritage studies

I confirm that I have read this submission and believe that I have an appropriate level of expertise to confirm that it is of an acceptable scientific standard, however I have significant reservations, as outlined above.

# Response

**Published:** 2025-05-02

**Authors:** Susanta Bahinipati

**Affiliations:** Film and Media Sciences, Kalinga Institute of Industrial Technology, Bhubaneswar, Odisha, India

**Reviewer- 1**

**Ngakan Ketut Acwin Dwijendra**, Udayana University, Denpasar, Indonesia

 The Konark Temple’s Construction: A Critical Review of the Historical, Cultural, and Scientific Evidence

 We are grateful for your appreciation of the work and constructive comments/ suggestions to improve the quality of the manuscript. 

**Summary of the Article**

 The article critically reviews the historical, cultural, and scientific evidence surrounding the construction of the Konark Temple in Odisha, India. The research incorporates data from ancient texts, folklore, palm leaf manuscripts, archaeological evidence, and historical records. It explores themes such as the temple's purpose, the techniques and materials used, the role of indigenous knowledge, and its architectural and astronomical precision.

 Key findings include:

- The temple served as a monument of devotion to the Sun God and a symbol of King Narasimhadeva I’s military victories.
- Advanced construction techniques included using rust-resistant iron, innovative stonelifting technologies, and precise architectural designs.
- The temple's alignment with astronomical phenomena demonstrates ancient artisans' mastery of astronomy.
- Indigenous technologies, including the use of high-grade iron and efficient transportation of massive stone blocks via sea routes, highlight the engineering ingenuity of the time.

The article concludes that the Konark Temple embodies a blend of artistic mastery, cultural significance, and technological innovation, offering insights for contemporary architecture and engineering.

**Response: **Thank you for your thoughtful and constructive feedback.

**Listed Reply to Comments/ Suggestions**

 Here follows the listed reply to comments/suggestions.

1. **Comprehensiveness of the Topic in Current Literature**

**Evaluation:** **Partly**. The article extensively reviews the historical and cultural significance of the Konark Temple, presenting a rich narrative of its construction and legacy. However, its engagement with contemporary research methodologies in archaeology, materials science, and geotechnical studies is limited. While the authors mention some modern studies, such as those by CSIR and CBRI, these are not deeply integrated into the analysis. For example, insights from recent archaeological technologies like ground-penetrating radar or 3D modeling could enhance the study’s relevance.

**Recommendations: **Incorporate more recent geotechnical or archaeological studies to substantiate claims about the temple’s construction techniques and materials. Review and discuss contemporary literature on similar historical structures globally for comparative insights.

**Response: **We have already included the geotechnical and archaeological studies as follows:

- **Ground-Penetrating Radar (GPR) Studies:**
  - Dwivedi et al. (2022) on heritage structure foundation investigation (discussed in the "Foundation of KT" section).
  - Jana et al. (2016) on palaeo-channel search near Konark (covered in "Introduction").
- **Multi-Disciplinary River System Analysis:**
  - Jana et al. (2022) on delineation of ancient rivers, providing insights into stone transportation (included in "Stone Quarrying and Transportation of Stones").

While detailed applications of ground-penetrating radar (GPR) and 3D modelling were not directly incorporated into this study, their potential use has been outlined in the second paragraph of the ‘Directions for research’ section for future investigations. Comparative insights on similar global historical structures have also been included, as recommended and problem is derived for further research.

 Second, geological and remote sensing studies have revealed that the Giza Pyramids in Egypt were once connected to now-defunct branches of the Nile, likely used to transport limestone during construction (Ghoneim et al., 2024; Sheisha et al., 2022). At Angkor Wat in Cambodia, Light Detection and Ranging (LiDAR) and Geographic Information System (GIS) technologies uncovered a sophisticated hydraulic infrastructure integral to the site's construction and urban planning (Evans et al., 2013; Uchida & Shimoda, 2013). In the UK, investigations at Stonehenge using Ground Penetrating Radar (GPR), Electromagnetic Induction (EMI), and 3D modelling revealed buried features and refined interpretations of the monument’s astronomical alignments (Gaffney et al., 2018). Similarly, at Chichen Itza in Mexico, archaeoastronomical studies of the Caracol Tower revealed its precise alignments with solstices and Venus cycles (Aveni et al., 1975). These interdisciplinary approaches demonstrate the power of technologies in decoding ancient engineering and cosmological knowledge. Applying similar methods, such as GIS, 3D modelling, and GPR can help uncover the historical mysteries surrounding KT’s sites, including the locations of ancient water bodies and ports, construction materials, logistical systems, astronomical alignments, and broader environmental interactions.

 To understand KT's construction and stone transport, soil analysis and borehole data can reveal ground conditions that shaped building techniques. Geophysical tools like GPR and Electrical Resistivity Imaging (ERI) can detect buried features such as ancient water channels. Satellite radar imagery and GIS help trace historical water bodies and landscapes by layering topography, hydrology, and old maps. Additionally, sediment coring, sonar, and bathymetric surveys using tools like sub-bottom profilers and remotely operated vehicles (ROVs) can explore potential maritime transport links. Metallurgical and archaeological studies can also test historical claims, such as the possible use of magnets in KT.

**References**

 Aveni, A. F., Gibbs, S. L., & Hartung, H. (1975). The Caracol tower at Chichen Itza: An ancient astronomical observatory? *Science, 188*(4192), 977–985. https://www.jstor.org/stable/1740708

 Evans, D. H., Fletcher, R. J., Pottier, C., Chevance, J. B., Soutif, D., Tan, B. S., ... & Boornazian, G. (2013). Uncovering archaeological landscapes at Angkor using LiDAR. *Proceedings of the National Academy of Sciences, 110*(31), 12595–12600. https://doi.org/10.1073/pnas.1306539110

 Gaffney, V., Neubauer, W., Garwood, P., Gaffney, C., Löcker, K., Bates, R., ... & Corkum, A. (2018). Durrington Walls and the Stonehenge Hidden Landscape Project 2010–2016. *Archaeological Prospection, 25*(3), 255–269. https://doi.org/10.1002/arp.1702

 Ghoneim, E., Ralph, T. J., Onstine, S., El-Behaedi, R., El-Qady, G., Fahil, A. S., ... & Fathy, M. S. (2024). The Egyptian pyramid chain was built along the now abandoned Ahramat Nile Branch. *Communications Earth & Environment, 5*(1), 233. https://doi.org/10.1038/s43247-024-01379-7

 Sheisha, H., Kaniewski, D., Marriner, N., Djamali, M., Younes, G., Chen, Z., ... &Morhange, C. (2022). Nile waterscapes facilitated the construction of the Giza pyramids during the 3rd millennium BCE. *Proceedings of the National Academy of Sciences, 119*(37), e2202530119. https://doi.org/10.1073/pnas.2202530119

 Uchida, E., & Shimoda, I. (2013). Quarries and transportation routes of Angkor monument sandstone blocks. *Journal of Archaeological Science, 40*(2), 1158–1164. https://doi.org/10.1016/j.jas.2012.12.018

  

1. **Factual Accuracy and Citation Support **

**Evaluation:** **Partly**. While the article is well-cited and draws on historical sources, its reliance on folklore and secondary sources raises questions about the verifiability of some claims, such as the legend of Dharmapada or the use of magnets to suspend objects. Some claims, like the rust resistance of the iron used, require more robust validation through referenced scientific studies.

**Recommendations: **Ensure that all key assertions, especially those based on folklore or oral traditions, are marked as such and distinguished from evidence-based claims. Include peer-reviewed scientific studies to corroborate statements about materials, such as the selection processes for rust-resistant iron and stone.

**Response: **The legends of Dharmapada and magnetic suspension are folklore and require scientific validation. This has been noted for future investigation in the third paragraph of the ‘Directions for research’ section, as stated in the following sentence.

 Metallurgical and archaeological studies can also test historical claims, such as the possible use of magnets in KT.

 To ensure robust validation, we have emphasized the need for peer-reviewed scientific studies on material properties, including rust-resistant iron. The discussion now integrates studies on ancient ironworking techniques and a comparative analysis of ironwork across India, which is detailed in the “Use of high-grade iron” section.

 Distinctions between historical evidence, oral traditions, and scientific analysis are reported below:

**Use of high-grade iron**

 The artisans of Konark demonstrated remarkable metallurgical expertise in shaping and assembling large iron beams over eight centuries ago. Engineer M.H. Arnott, who worked on early 20th-century excavations and restorations, documented their manufacturing techniques. Upon examining a broken iron beam, he discovered that the iron was forged in small segments, approximately 1 to 1.5 feet in length and 3 to 4 inches in width, and arranged in a staggered pattern similar to bricks in a wall (O’Malley, 1908). These segments were inserted into a hollow quadrilateral iron bar and welded together, ensuring a seamless and durable structure. The final polishing concealed any visible joints, resulting in a robust beam with mechanical properties comparable to contemporary military-grade steel (Edwards, 1969; Friend, 1926; Singh & Kaur, 2014).

 A metallurgical analysis by Friend (1926) compared Konark iron (c. 1250 CE) with other notable ancient ironworks, including the Delhi Iron Pillar (c. 300 CE), the Dhar Pillar (c. 320 CE), and Ceylonese (Sigiriya) Iron (c. 450 CE). The study revealed significant differences in composition, hardness, and corrosion resistance. Konark iron had a low phosphorus content (0.015%), whereas the Delhi Iron Pillar (0.114%), Dhar Pillar (0.28%), and Ceylonese iron (0.34%) contained higher amounts, which contributed to their superior corrosion resistance. The carbon content in Konark iron (0.110%) was slightly higher than that of the Delhi Iron Pillar (0.080%), but its higher sulphur content (0.024%) made it more brittle. Brinell hardness tests indicated that Konark iron (72) was softer than the Delhi Iron Pillar (188).

 Despite these findings, Friend’s controlled experiment revealed that Konark iron exhibited remarkable durability. Identical samples of Konark iron and modern military-grade steel were exposed to alternating wet and dry conditions for one year, followed by submersion in artificial seawater for another year. The results showed that modern mild steel deteriorated completely, whereas Konark iron retained 89.3% of its original structure. Furthermore, under artificial seawater exposure, modern mild steel corroded extensively, while Konark iron preserved 75.3% of its integrity.

 These findings affirm the advanced metallurgical knowledge of ancient Indian artisans. Their ability to produce durable iron structures is superior to the twentieth century engineers.

 These findings affirm the advanced metallurgical knowledge of ancient Indian artisans, whose ability to produce durable iron structures surpassed that of many twentieth-century engineers. Ghosh (1963) provides a detailed metallurgical analysis of the Delhi Iron Pillar, emphasizing its high phosphorus content and the absence of manganese—key factors contributing to its exceptional corrosion resistance. The pillar was constructed through forge welding of small iron blooms, a technique similar to that used for the Konark iron beams. While the Delhi Iron Pillar benefited from a dry climate that minimized rust formation, the Konark beams are regularly and harshly exposed to humidity and salt-laden winds on the seacoast. Despite these challenges, the Konark iron beams have endured for centuries, indicating that the artisans employed advanced forging and welding techniques to enhance durability. Ghosh’s comparison highlights the ingenuity of ancient Indian artisans in adapting techniques to counter specific structural degradation. The enduring presence of iron beams at Konark, despite centuries of exposure to a harsh coastal climate, attests to the artisans’ sophisticated craftsmanship. The fusion of indigenous knowledge and technical proficiency continues to astonish researchers worldwide, reinforcing the ingenuity of ancient construction techniques in ensuring structural durability.

 Reference:

 Ghosh, M. K. (1963). The Delhi iron pillar and its iron. *NML Technical Journal*, *5*(1), 31-45.

1. **Accessibility of Language**

**Evaluation:** **Yes**. The language is clear, engaging, and accessible to academic and general audiences. The integration of technical and cultural narratives is particularly commendable.

**Recommendations: **Maintain this clarity while adding more technical terms and methodologies if engaging with geotechnical or engineering audiences.

**Response: **Thanks. We appreciate your acknowledgment of the clarity and engagement of the language.

1. **Appropriateness of Conclusions**

**Evaluation:** **Partly**. The conclusions are generally appropriate, synthesizing the findings effectively. However, they are somewhat limited by the scope of the evidence presented. For example, the findings could better integrate critiques or limitations of ancient techniques compared to modern methodologies.

**Recommendations: **Discuss how the findings contribute to or challenge current historical architecture and engineering knowledge. Acknowledge gaps in evidence and propose specific areas for future research.

**Response: **We have refined the conclusion section to integrate ancient techniques and their relevance to modern engineering.

**Conclusion**

 This study highlights the remarkable expertise of the artisans involved in constructing the KT. By examining folk narratives, cultural practices, and sculptural evidence, we gain deeper insights into the historical and technological advancements that shaped this architectural marvel. Analysing ancient construction techniques enhances our understanding of past engineering methods while offering valuable lessons for modern architects and engineers seeking sustainable and resilient design solutions.

 Our research underscores the enduring legacy of Narasimhadeva I, as reflected in the temple’s precise measurements, originally recorded in the Sanskrit *Madalapanji*, which align with modern calculations. The study also sheds light on the logistical ingenuity of ancient artisans, particularly their use of maritime routes to transport massive stone blocks. Additionally, their selection of high-grade stones, resistant to erosion, demonstrates their advanced understanding of material durability. Furthermore, sculptural evidence reveals historical anecdotes, such as the valour of Sudehi, the faithful elephant, illustrating the intersection of history and legend in the temple’s narrative.

 Geotechnical evaluations validate the stability of the temple’s foundation in a coastal environment, reinforcing the feasibility of its architectural blueprint. Furthermore, the study reveals sophisticated construction techniques used to lift enormous stone blocks to great heights. The use of iron scaffolding, akin to modern construction methods, highlights parallels between ancient and contemporary engineering practices.

 A significant finding is the presence of rust-resistant iron beams within the temple compound, an aspect that continues to intrigue metallurgists and material scientists. However, while this study presents compelling evidence of advanced metallurgical knowledge, further analysis is needed to fully understand the composition and long-term durability of these materials. The temple’s wheels, serving as India’s earliest sundial, along with its astronomical alignments, attest to the artisans’ deep understanding of celestial mechanics, particularly in channelling the first rays of the sun into the inner sanctum.

 While this study consolidates historical, cultural, and scientific evidence surrounding the KT, it also acknowledges certain limitations. The absence of direct archaeological excavations and detailed material testing limits the verification of oral narratives. Future research should focus on metallurgical studies, geospatial analysis, and experimental reconstructions to further investigate construction techniques and environmental interactions. By bridging ancient and modern technologies, this study not only unfolds the 13th-century technological ingenuity but also opens new avenues for interdisciplinary research into the temple’s enduring mysteries.

**Specific Points for Scientific Soundness**

1. **Integration with Current Research**

**Include recent studies or methods, such as geotechnical assessments or 3D modeling, to validate claims about construction techniques. Provide a comparative analysis with other ancient monumental structures to position the findings in a broader context.**

**Response:**We appreciate the suggestion to incorporate recent methodologies. The manuscript integrates **geotechnical and geophysical studies**, including **GPR** and **remote sensing**, to analyze the **structural and environmental context** of the KT. These aspects are detailed in the section **“Foundation of Konark Temple.”** Additionally, the **“Directions for Future Research”** section highlights the potential applications of **3D modeling, GIS-based analysis, and advanced geotechnical investigation techniques.**

 To strengthen **interdisciplinary connections**, we have expanded the **comparative analysis** of construction techniques in **other ancient monumental structures** such as the **Giza Pyramids, Angkor Wat, Stonehenge, and Chichen Itza.**

**Strengthening Evidence**

 Use scientific data to support claims about material properties, such as the corrosion resistance of iron used in the temple. Provide citations from peer-reviewed journals, especially for claims about astronomical alignment and structural engineering.

**Response: **We have expanded the **"Use of High-Grade Iron"** section to include **metallurgical analyses comparing Konark iron with other ancient iron structures** such as the **Delhi Iron Pillar, Dhar Pillar, and Sigiriya Iron**. This discussion now incorporates **data on chemical composition, hardness, and corrosion resistance**, citing relevant **peer-reviewed studies** (e.g., Ghosh, 1963; J. Newton Friend, 1926; Singh & Kaur, 2014).

 For **astronomical alignments**, we have integrated **scientific references** to substantiate claims regarding **the temple’s wheels functioning as a sundial** and its **architectural design channeling the sun's first rays into the sanctum**. These citations (e.g., Bhatnagar & Livingston, 2005; Das, 2015; De, 2022; Dey, 2016; John *et al*., 2015; Joshi & Srivastava, 2021; Yadav, 2021) ensure that all **astronomical interpretations are based on scientific validation rather than conjecture**.

1. **Distinguishing Between Fact and Folklore**

**Differentiate between historical records, archaeological evidence, and folklore. For example, legends like the story of Dharmapada should be framed as cultural narratives unless supported by evidence.**

**Response: **We appreciate the suggestion to distinguish between **historical records, archaeological evidence, and folklore**. We have discussed the **Dharmapada legend** to clearly separate cultural narratives from verifiable historical sources.

- The **"Recollections of Construction and Human Resources"** section explicitly categorizes the **story of Dharmapada** as an **evolving folklore**, shaped over centuries through oral traditions, nationalist influences, and literary adaptations (e.g., O’Malley, 1908; Mishra, 1919; Das, 1924).
- To provide **historical grounding**, we incorporate **palm leaf manuscript references (Boner, 1970; Boner et al., 1972)** that mention **Dharma Mahapatra as a real historical figure** involved in the temple’s construction, ensuring a **fact-based distinction** between folklore and documented accounts.
- We acknowledge that while **folk traditions convey cultural values**, they should not be conflated with empirical evidence. The text **contextualizes **Dharmapada’s story within the broader **socio-political and artisan-based reality** of the temple's construction.

By adopting this approach, we ensure that **folklore is presented as part of Konark’s cultural legacy**, while historical claims remain **firmly supported by documented sources**.

  

1. **Clarification of Methodology**

Elaborate on the systematic review process, specifying how documents were selected and analyzed. Discuss potential biases in the sources.

**Response: **We have elaborated on the systematic review process in the “ **Methodology”** section, detailing document selection, analysis, and potential biases in the sources. Additionally, the **structure of the paper** has been presented in **Figure 1** for clarity. The changes are as follows:

**A systematic review methodology was adopted for the search and selection of documents. Documents were sourced from Scopus, the largest scientific journal database, and Google Scholar, an inclusive, automated repository offering access to a vast range of published works (Bar-Ilan, 2008). Two search strings were employed: “KONARK” OR “SUN AND TEMPLE” within the title, abstract, and keyword fields. This search yielded 329 documents from Scopus and 912 from Google Scholar. After removing duplicates (n = 235), the remaining documents were screened on the basis of titles and/or abstracts for relevance to nine thematic areas: (1) causes of construction, (2) financial allocation, (3) dimensions of Konark Temple (KT), (4) human resources, (5) obstacles during construction, (6) foundation of KT, (7) stone selection, transportation and lifting, (8) native technology, and (9) astronomical phenomena. This process resulted in 80 documents, comprising 29 journal articles and 51 books, and excluded 926 documents. Following a full-text review, 69 documents, containing 29 journal articles and 40 books, were retained as relevant to the aforementioned themes, while 11 books were excluded by the first author. The inclusion and exclusion of documents were subsequently discussed among all three authors, and the exclusion was agreed upon.**

**Limited programmatic research and publications in journals on the KT resulted in the retention of 29 journal articles. As KT is an ancient structure dating back to the 13th century, 36 documents related to its architecture, construction, water bodies, and associated folklore were selected from cross-referenced old books and ancient writings (n = 29), palm-leaf inscriptions and indigenous narratives on stones (n = 6), and copper plates (n = 1). Also, 13 journal articles were selected for a comparative analysis of KT with other monumental structures worldwide (Aveni, Gibbs, & Hartung, 1975; Evans et al., 2013;** **Farhan et al., 2022; Gaffney et al., 2018; Ghoneim et al., 2024; Sheisha et al., 2022; Uchida & Shimoda, 2013), for assessing the quality of stone and iron used (Basu, Orr, & Aktas, 2020; Church of England, 2019; Ghosh, 1963; Gilchrist, 2001; Phys.org, 2012; Vincent, 1993). An additional journal article was included to rationalise the database for selection and inclusion of documents for review (Bar-Ilan, 2008). These were initiated by the first author and agreed upon by the remaining two authors, with no documents removed. Of 130 documents, 119 documents were included for this review (see Fig. 2). The inter-rater reliability (Number of agreed documents/ Total number of documents) was 91.54%.**

  

1. **Addressing Limitations**

Acknowledge the study's limitations, such as the reliance on secondary sources and the lack of direct archaeological validation for some claims.

**Response: **We acknowledge the study's limitations, particularly the reliance on secondary sources and the lack of direct archaeological validation for some claims. These constraints have been addressed in the **Limitations** section as follows: 

**Limitations**

 The limited availability of journal publications on the KT constrains access to authentic, peer-reviewed evidence. Nevertheless, this study relies on archival data derived from indigenous knowledge dating back to the 13th century, incorporating folk narratives and prevailing cultural practices. Documentation includes incidents and eyewitness accounts recorded in books, diaries, palm leaf manuscripts, and stone inscriptions.

 Furthermore, the study examines sculptures from the KT, as well as those housed in museums across various locations, alongside an exploration of the ancient maritime traditions of the kingdom’s predecessors. Literature authored by King Narasimhadeva I’s court poet and historical records of the kingdom also provide valuable insights.

 Additionally, the study acknowledges its reliance on secondary sources, which may introduce potential biases in historical interpretations. While efforts have been made to validate these sources through cross-referencing with archaeological records, geophysical surveys, and metallurgical analyses, the absence of direct archaeological validation for certain claims remains a constraint. To address this, future research may adopt a multidisciplinary approach integrating geotechnical, geophysical, and material analyses for a more comprehensive understanding.

**Conclusion**

 Overall, the article offers valuable insights into the construction of the Konark Temple and highlights its historical, cultural, and scientific significance. The authors should address the abovementioned limitations to make the article scientifically sound, particularly by integrating more recent research, strengthening evidence-based claims, and distinguishing folklore from verifiable facts. By doing so, the study can serve as a robust reference for both historical and scientific investigations into ancient construction techniques.

**Response:** We appreciate the constructive feedback on our manuscript. In response, we have addressed the identified limitations by:

- Integrating more recent research and peer-reviewed studies to strengthen evidence-based claims.
- Distinguishing folklore from verifiable historical facts by clearly categorizing narratives within the text.
- Expanding the methodology section to enhance transparency regarding source selection and validation.

Regarding the comprehensive discussion of the topic, the study incorporates a multidisciplinary approach, integrating historical records, indigenous knowledge, metallurgical analyses, and geophysical insights. While the existing literature on the KT remains limited, we have ensured that the discussion aligns with current research findings and emerging perspectives on ancient construction techniques.
